# Cell Therapy for Anal Sphincter Incontinence: Where Do We Stand?

**DOI:** 10.3390/cells10082086

**Published:** 2021-08-13

**Authors:** Alexandre Balaphas, Jeremy Meyer, Raphael P. H. Meier, Emilie Liot, Nicolas C. Buchs, Bruno Roche, Christian Toso, Leo H. Bühler, Carmen Gonelle-Gispert, Frédéric Ris

**Affiliations:** 1 Division of Digestive Surgery, University Hospitals of Geneva, 1205 Geneva, Switzerland; jeremy.meyer@hcuge.ch (J.M.); emilie.liot@hcuge.ch (E.L.); nicolas.c.buchs@hcuge.ch (N.C.B.); bruno.roche@grangettes.ch (B.R.); christian.toso@hcuge.ch (C.T.); frederic.ris@hcuge.ch (F.R.); 2 Department of Surgery, Geneva Medical School, University of Geneva, 1205 Geneva, Switzerland; 3 Department of Surgery, University of Maryland School of Medicine, Baltimore, MD 21201, USA; RMeier@som.umaryland.edu; 4 Faculty of Science and Medicine, University of Fribourg, 1700 Fribourg, Switzerland; leo.buhler@unifr.ch (L.H.B.); carmen.gonelle@unifr.ch (C.G.-G.)

**Keywords:** anal incontinence, fecal incontinence, stem cell, progenitor cell, mulitipotent cell, myoblast, satellite cell, mesenchymal stem cell, mesenchymal stromal cell

## Abstract

Anal sphincter incontinence is a chronic disease, which dramatically impairs quality of life and induces high costs for the society. Surgery, considered as the best curative option, shows a disappointing success rate. Stem/progenitor cell therapy is pledging, for anal sphincter incontinence, a substitute to surgery with higher efficacy. However, the published literature is disparate. Our aim was to perform a review on the development of cell therapy for anal sphincter incontinence with critical analyses of its pitfalls. Animal models for anal sphincter incontinence were varied and tried to reproduce distinct clinical situations (acute injury or healed injury with or without surgical reconstruction) but were limited by anatomical considerations. Cell preparations used for treatment, originated, in order of frequency, from skeletal muscle, bone marrow or fat tissue. The characterization of these preparations was often incomplete and stemness not always addressed. Despite a lack of understanding of sphincter healing processes and the exact mechanism of action of cell preparations, this treatment was evaluated in 83 incontinent patients, reporting encouraging results. However, further development is necessary to establish the correct indications, to determine the most-suited cell type, to standardize the cell preparation method and to validate the route and number of cell delivery.

## 1. Introduction

Anal sphincter incontinence (ASI) is defined by an involuntary loss of gases, solids and/or liquid materials. The prevalence of ASI is widely underestimated and ranges from 0.4 to 24% [1]. ASI is an underrated pathology in regards to its morbidity and its impact on the society and is associated with impaired quality of life and psychopathologies such as depression, anxiety disorder and impaired sexual function [2]. In the USA, ASI’s global cost has been estimated to be around 11 billion dollars per year [2].

The physiopathology of ASI is not completely understood but results from a functional impairment of one or more of the four anatomic components that allow a normal continence: The internal anal sphincter (IAS), the external anal sphincter (EAS), the pelvic floor muscles and the sensory-motor apparatus [3]. ASI is primarily managed conservatively but sphincter repair surgery is proposed for large defects of either EAS, IAS or both with conservative treatment failure [4,5]. However, long-term results of anal sphincter repair are unsatisfactory, notably regarding IAS repair [6,7,8,9]. Several strategies have been developed to improve sphincter repair surgery or to replace it without outstanding successes [10,11,12]. Since more than a decade, the transplantation of stem cells (multipotent cells with limited proliferation ability) or progenitor cells (multipotent cells with limited proliferation ability) has been proposed as an adjunct or direct therapy for ASI following the hypothesis that transplanted cells will either commit differentiation into EAS or IAS muscle or produce growth factors that will favor sphincter healing (Table S1). A consensus among researchers was never reached, and various source cells and protocols have been proposed and investigated with preclinical and clinical studies. We present herein a critical analysis of cell therapy for ASI. Of note, as transplanted cells were heterogenous regarding their origin and phenotype, the generic term “multipotent cell” will be used to describe the transplanted cells.

## 2. Physiopathology of Anal Sphincter Injuries, Models and Cell Therapy-Based Strategies

### 2.1. Mechanisms of Sphincter Injuries

In patients with ASI, there are at least two mechanisms leading to a modification in the pelvic floor structure or function. First, sphincters can be injured by perineal tearing, stretching and/or ischemia; obstetric trauma being the most common cause [13]. The episiotomy procedure is also associated with anal incontinence [14]. A second mechanism involves the degeneration of pelvic floor muscles, which appears with ageing, but rapidly after menopause as pelvic floor structures are hormone responsive [13]. Proposed animal models of anal incontinence tried to simulate distinct clinical situations where continence is impaired by an acute or chronic mechanism. In these models, various methods were used to harm these structures, including muscle section or excision, thermal injury and even nerve crushing [15] (Table 1). However, these experimental models of ASI do not consider the stretching effect of pelvic floor that can occur during childbirth. Thus, some authors have proposed a more realistic rodent model of childbirth injury by means of a balloon inflated in the vagina for a prolonged time [16,17]. This model was, however, never used to assess the potential of cell therapy to cure ASI.

### 2.2. Acute Anal Sphincter Injury and Healing

**Figure 1:**
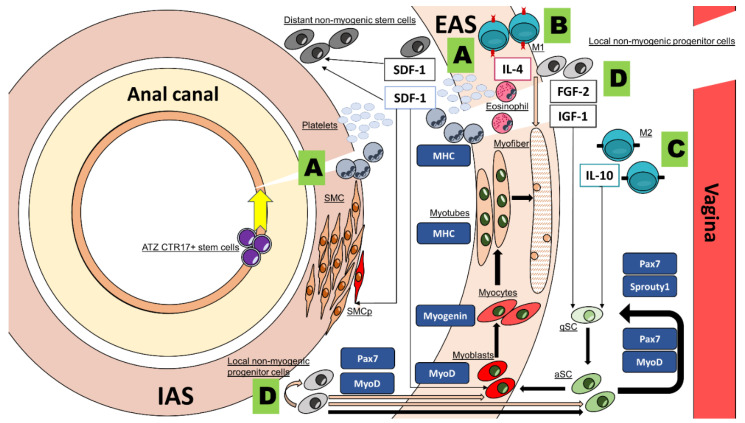
Schematic representation of events occurring after delivery-related acute anal sphincters injury [51,53]. (**A**): The tear will induce a phase of inflammation where polymorphonuclear cells are attracted in the first hour and are responsible to maintain the function of macrophages that will arrive later. At the same time, platelets recruitment following the rupture of small vessels induces the releasee of SDF-1 following their degranulation. SDF-1 is primordial for anal sphincter regeneration and acts by attracting non-resident progenitor cells, myoblasts and smooth-muscle progenitors to the wound and by promoting the survival, migration and proliferation of these cells. (**B**): Macrophages of M1 (CD68^+^) polarity arrives after the polymorphonuclear cells and largely contribute to the acute inflammation phase. (**C**): During the healing phase, activated satellite cells contribute to new muscle formation (black arrows) and each step is characterized by a specific hallmark protein expression (blue boxes). Then, macrophage polarity switches to M2 (CD163^+^) and they produce cytokines such as Il-10 helping proliferation and differentiation of satellite cells. (**D**): During the healing phase, different kinds of non-myogenic local progenitor cells are activated, and some of them, like the fibro-adipogenic progenitors, are conditioned by the presence of IL-4 secreted by eosinophils, with this pathway being necessary for skeletal muscle regeneration. Non-myogenic local progenitor cells interplay indirectly by the production of cytokines like FGF-2 and IGF-1 but can also fuse (camel arrows) with other non-myogenic local progenitor cells, satellite cells, myoblasts or even myofibers, inducing hypertrophy. Finally, in case of damage to the rectal wall mucosa, local stem cells of the anal transition zone contribute to the reparation of the rectum and anal transition zone mucosa. qSC: Quiescent satellite cell, aSC: Activated satellite cell, SMC: Smooth muscle cell, pSMC: Progenitor smooth muscle cell. ATZ: Anal transition zone.

The classical clinical situation of acute anal sphincter injury is well illustrated by childbirth trauma, which still occurs in 11% of vaginal deliveries and can extend up to the IAS and sometimes the rectum (starting from posterior wall of vagina) [49] (Figure 1). In the case of extending traumatism to the level of rectal wall, dedicated stem cells of the anal canal transition zone, positive for cytokeratin 17, participate in the healing process of the mucosa of the rectum and the anal canal [50]. On the other hand, the healing process of anal sphincters has not been thoroughly studied and it is supposed to be very similar to other muscle-healing processes [51]. Under optimal conditions, healing ultimately leads to the generation of new myofibers/smooth muscle cells from muscle satellite cells/progenitor smooth muscle cells or the reparation of damaged myofibers after fusion with muscle satellite cells [52,53]. After anal sphincter injury, it is thought that inflammatory cells’ cross-talk produces cytokines and growth factors that will recruit stem cells and progenitor cells [51,53]. These cells could be mobilized from bone marrow or the surrounding tissues and might favor healing, be incorporated into the wound or further recruit stem cells and progenitor cells [51,53,54]. Especially, skeletal muscle satellite cells, which are localized in periphery of myofibers, near vascular or nerve structures, are a source of myoblasts and, further, new myocytes after their activation [55,56]. However, Lorenzi et al. reported in rats, after direct anal sphincter injury and repair, the persistence of fibrous tissue with dilated blood vessels and muscle cell degeneration patterns [19].

Progenitor cells produce cytokines like stromal-derived factor 1 (SDF-1) that are both chemoattractive for progenitor cells (including myoblasts and smooth muscle progenitor cells) but also contribute to cell proliferation, migration and survival [51,57,58]. SDF-1 seems to be a prominent cytokine for anal sphincter healing. Salcedo et al. reported a rapid local burst of SDF-1 and monocyte chemotactic protein-3 (MCP-3) expression in rats one hour after sphincter injury and up to 21 days after injury [59]. Moreover, an injection of plasmids with SDF-1 directly into EAS muscle or transplantation of SDF-1 transfected progenitor cells both improved continence in rodents after partial sphincterectomy with the same extend [35,36]. 

**Figure 2:**
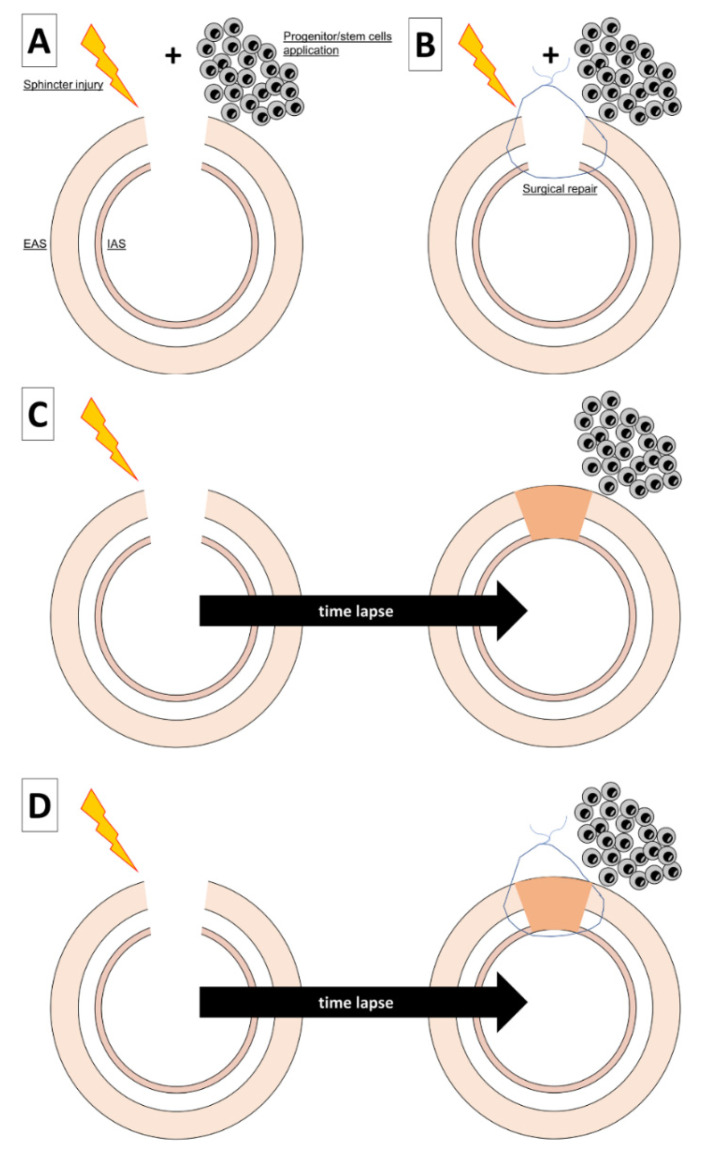
Cell therapy-based strategies for anal sphincter incontinence treatment. (**A**,**B**) Multipotent cells can be transplanted directly after injury with or without simultaneous reparation. (**C**) After injury, healing progresses but sometimes the muscle tissue is altered and responsible for ASI. Multipotent cells can be transplanted directly into the scar or in the neighborhood. (**D**) The best scenario could be to reinforce the surgical reparation of a sphincter defect with multipotent cells.

In theory, progenitor cells could be transplanted soon after injury and this is considered to be the best option to maximize the effect of transplantation (Figure 2) [51]. Indeed, it is supposed that progenitor cells might increase the natural healing process notably through the local release of cytokines such as SDF-1 [51,59]. This strategy was evaluated in several preclinical studies where progenitor cells were either directly injected after injury with surgical reparation, or not, to mimic the situation of a direct repair of sphincters [18,19,20,21,22,23,24,25,32]. Of note, some authors delayed this intervention by 24 h [15,31,32,38]. To the best of our knowledge, progenitor cells have never been injected in a patient with acute anal sphincter injury.

### 2.3. Unrepaired Anal Sphincter Injury

Most often, an anal sphincter tear is usually identified after delivery and surgically repaired [49]. Sometimes, the diagnosis can be missed, resulting in an occult anal sphincter injury [60]. As for an acute anal injury, little is known about the long-term healing and remodeling of damaged muscle, and an analogy can be made with the repair processes of skeletal and smooth muscles from other localizations (cf. Section 2.2). Probably, some unrepaired anal sphincter lesions could spontaneously heal. There exists evidence that a clear cut through anal sphincters in rats can heal spontaneously without inducing ASI [31,39]. As a result, almost all models of ASI in rats imply the partial resection of anal sphincters. In rat models where simple anal sphincters section were performed, acute and chronic inflammation was seen at the site of injury, characterized by neutrophils, monocytes macrophages infiltrations and fibrous tissue [20,22], disorganization of striated fibers of the EAS [35,37], but also mucin pool inclusions with histiocytes [34]. In rabbits, Rajasekaran investigated the effects of an EAS clear-cut section over time. The authors reported early collagen deposits from one week after injury, but also extensive fibrosis appearing three weeks after injury, at the site of myotomy but also beyond [61]. The presence of fibrosis was confirmed by other authors in rabbits [40,43]. In dogs, three weeks after IAS and EAS partial excision, Kang et al. reported focal interstitial inflammation, fibrosis and atrophy of smooth and striated muscles [46].

Anal sphincters encircle the anal canal, and the loss of this circular shape, as a result of injury, directly impairs the continence function [62]. However, during sphincter repair surgery, one can observe that the retracted muscle edges are held together with fibrous tissue, which bridges the defect [63]. Thus, unrepaired damage to IAS or EAS does not evolve into a hole in the sphincter ring (EAS and/or IAS), but rather into altered tissue, which fills the breach. In rats, this tissue contained mast cells and other inflammatory cells [18]. To the best of our knowledge, the importance of this tissue was never investigated in humans but could be of importance, especially if the muscle gap is the target of cell therapy.

In the majority of preclinical studies, researchers investigated the direct injection of mesenchymal stem cells (MSC) in the area of muscle injury (Figure 2). This approach could be wrong as recent evidence stated that the transplantation of stem cells of mesenchymal origin during the late phase of healing can be deleterious and increase fibrosis [64]. Surprisingly, in the majority of fundamental studies modeling anal injury, the recovery time before cell therapy was very short (days) whereas after sphincter injury, ASI appears years or even decades after the initial trauma [65]. Therefore, future models should also consider a realistic healing time after anal sphincter injury before applying cell therapy. Indeed, trials with patients suffering from long-lasting ASI with EAS lesions have investigated the direct injection of multipotent cells into EAS lesions [66], muscle edges [66,67] or entire muscle [68,69].

Another strategy to treat patients with ASI is the transplantation of a biosphincter. Bitar’s group, from the Wake Forest Institute for Regenerative Medicine, has been working for decades on the implantation of bioengineered anal sphincters composed of innervated smooth muscle cells (Table S2). The results are promising and the group recently implanted the construct in large animals, including non-human primates [47,48]. This strategy is interesting as it provides a complete and functional IAS substitute and might be part of a reconstruction strategy for patients in whom IAS has been totally or partially removed such as for ultra-low rectal cancer resections. However, IAS engineering has some hurdles to overcome. First, this approach uses IAS or digestive smooth muscle cells and intestinal neuronal cells. For translation to the clinic, tissue might be procured from organ donors, requiring further immunosuppression and exposing patients to its associated risks. Those risks need to be balanced with the fact that ASI is a non-life-threatening condition. Moreover, Araki et al. recently demonstrated the feasibility of anorectal transplantation in a dog model, with this approach being serious concurrent to allogenic biosphincters [70]. Further, the tissue culture of such constructs requires 6 to 8 weeks of culture, which increases the risk of microbial contamination. Finally, the cost of the creation of an organic construct seems to be higher than cell isolation and expansion.

### 2.4. Secondary Repaired Anal Sphincter Injury

Patients with visible EAS or IAS sphincter lesions are good candidates for surgical repair. Park’s sphincteroplasty is the most common procedure where the two edges of the damaged sphincters are brought together with an overlapping suture [5,63]. Usually, the surrounding fibrous tissue, which also connects the retracted muscle, is not dissected as it offers a firm support for knotting [63]. To the best of our knowledge, the healing of such a delayed repair has never been investigated. Due to the poor long-term results of sphincteroplasty, the idea to strengthen the reparation with multipotent cells has emerged. As mentioned above, in preclinical studies, some authors injected multipotent cells during sphincter repair surgery but this was never done after secondary repair in animals [20,21,24,25,33]. However, Sarveazad et al. successfully and safely injected adipose tissue-derived multipotent cells after EAS sphincteroplasty in five women and two men (Table 2) [71].

### 2.5. Chronic Anal Sphincter Impairment

In some patients with ASI, a clear injury event cannot be found, and patients have either weak anal sphincter tonus without an anomaly on imaging or hypotrophy of anal sphincters on imaging. This could be the result of aging, denervation or degenerative mechanisms inducing a chronic injury to the anal sphincters [39]. IAS is responsible for the anal sphincter basal tone and contributes to up to 85% of the pressure necessary to keep the anal canal closed [75]. Indeed, IAS basal tonus is maintained without neural stimulus by the high expression of proteins inducing contractility in the IAS smooth muscle cells [76,77]. A high content of smooth muscle actin and myosin isoforms has been reported in IAS [77,78,79], but spontaneous contraction property is driven by the continuous phosphorylation of myosin light chain by protein kinase C (PKC) and Rho kinase systems; indeed proteins of these cascades, such as PKCα, RhoA, CPI-17 and HSP27 were prominent in human IAS smooth muscle cells compared to smooth muscle cells sampled from a human colon used as a control [79]. Specifically, it was demonstrated in humans that the RhoA/ROCK system is the main effector of the IAS basal tone and is constitutively activated by the renin-angiotensin system and arachidonic acid products [80]. On the other hand, IAS non-adrenergic non-cholinergic nerves negatively regulate the basal tone, essentially through nitric oxide (NO), which is the main neurotransmitter negatively affecting IAS [81]. Moreover, it was recently demonstrated in rats that RhoA/ROCK system proteins were the target of microRNA, which were reducing the IAS basal tone by a diminution of the expression of components necessary for signal transduction [82,83]. Particularly, micro-RNA-133a was increased with aging in rats and might partially explain the degeneration of IAS [82]. Other effects of aging, observed in colon smooth muscle cells, could also be present in IAS, including a diminution of RhoA translocation, a diminution in the association of the pairs actin/myosin and tropomyosin/HSP27 and a decrease in the phosphorylation of HSP27 [84]. These elements could partially explain the weakness of anal sphincters seen in older people.

Diabetes has been associated with ASI [85] and may affect anal sphincters from the cellular to the nervous level [77,82,83]. However, the precise mechanism of diabetes on anorectal function has been poorly investigated. At the cellular level, the expression of miRNA-133, linked to IAS degeneration, was also increased in diabetic rodents [82]. Diabetic neuropathy affects gastrointestinal motility [86] and measures of anal function in diabetic patients showed a reduction of maximal squeeze pressure (which is a reflection of EAS function) in diabetic patients compared to healthy controls [87]. Furthermore, patients suffering from inflammatory bowel disease are more prone to report ASI. There is probably an overlap between the alteration in bowel motility, the decrease in rectal compliance due to rectum inflammation and the sequel of repeated anal fistula surgery [88,89]. However, further investigation of muscle alteration during inflammatory bowel disease has never been carried out. 

To date, there is unfortunately no model mimicking a degeneration of the muscles of EAS or IAS. Hosokawa et al. proposed a model of EAS and IAS injury using cardiotoxin but the injury was finally similar to sphincter resection [90]. This model was never used to assess cell therapy efficacity/efficiency.

## 3. Multipotent Cell Origin and Isolation

### 3.1. Multipotent Cell Origins

Multipotent cells proposed for ASI cell therapy can be derived from various tissues. The most frequent sites were skeletal muscle, bone marrow or adipose tissue. Cell preparations were either syngeneic or autologous, and allogeneic or xenogeneic transplantation was marginal (four preclinical studies and one clinical study). Cells from a muscular origin were used in the majority of the identified publications: In 17 studies, they originated from skeletal muscle or EAS [24,25,28,29,41,42,44,45,46,66,67,68,69,74,91,92,93] and in 11 studies, from smooth muscle or IAS [48,79,94,95,96,97,98,99,100,101,102] (Table 3). Four out of six clinical trials used skeletal muscle multipotent cells whereas the other used adipose tissue multipotent cells [66,67,68,69,74]. Two in vivo publications reported the use of commercial H9c2 rat heart myoblasts [20,34].

Cells of bone marrow origin were used in nine studies [15,18,19,22,31,33,36,38,40] and only six tested cells originating from adipose tissue [23,26,27,30,71,72]. Neural cells were used for bioengineered constructs in eight publications [95,96,97,98,99,102]. Bioengineered constructs used smooth muscle seeded with neuronal cells from different origins. Finally, only one publication evaluated the potential of human umbilical cord matrix cells [40].

Skeletal muscle-derived cells seem to be an interesting source for ASI therapy. Different multipotent cells can be extracted from skeletal muscle: Satellite cells and other resident multipotent cells, the former having the ability to become new satellite cells or myoblasts, the precursors of myocytes (Figure 1) [104]. Thurner et al. demonstrated that smooth muscle cells can eventually be derived from myogenic progenitors [105]. The function of other muscle resident multipotent cells is not completely understood and this category encompasses different kinds of multipotent cells such as adult pericytes, PW1+ interstitial cells and fibro-adipogenic progenitors [106].

Satellite cells have the ability to reform muscle fiber [104] and have been proposed to treat conditions such as Duchenne disease [107], coronary artery disease [108] and also urinary incontinence [109]. It is supposed that skeletal muscle multipotent cells differentiated into new myocyte and could resupply EAS with new fibers (Figure 1). Indeed, the apparition of new fibers in EAS and the expression of muscle proteins have been observed by several authors [28,42,44,45,91,92]. However, the appearance of new muscle fibers does not necessarily indicate that anal function is improved. In this regard, several in vivo studies [20,29,46] using myogenic cells were inconclusive concerning anal function recovery despite cell engraftment confirmation.

Cells derived from adipose tissue constitute an interesting option, as subcutaneous fat tissue is easily accessible. The cells used in the reported studies [23,26,27,71] had the characteristics of MSC including the ability to differentiate into various tissues. MSC are multipotent cells that have been evaluated over the last years to treat various conditions including spinal cord injury, corneal or uvea injury, lung injury, cerebral injury, colitis, alopecia, muscular degenerative disease, myocardial infarction, liver injury, multiple sclerosis, Parkinson disease, cancer and to improve wound healing [110]. Indeed, allogeneic adipose tissue MSCs became popular because of their poor immunogenicity and their availability after liposuction surgery. However, recent evidence demonstrated the development of donor-specific antibodies, and MSC rejection has been documented [111]. Besides MSC’s ability to differentiate into various cells, their paracrine action have been proposed to mediate most of their effects [110,112]. MSC produce a large amount of growth factors and extracellular vesicles [113]. Strategies using encapsulated MSC conserve the effects of MSC, notably on liver fibrosis, confirming the efficacy of paracrine action and treatment [114]. Thus, MSC can be seen as in situ bioreactors delivering growth factors to neighboring cells. MSC have the ability to induce smooth muscle regeneration from the gut and the bladder [115,116] and also skeletal muscle regeneration [117,118]. Thus, MSC therapy may promote the healing and regeneration of both IAS and EAS.

### 3.2. Methods for Multipotent Cell Isolation and Processing

Stem cell and progenitor cells were retrieved from rats, mice, rabbits, non-human primate or humans (Table S1). Different harvest methods were used according to the origin of the collected tissue, but protocols were similar to existing standard, with the first step of washing and decontamination followed by the digestion of tissue and, finally, the purification of the cell suspension before plating. In the majority of studies, isolation procedures were sufficiently detailed, but some studies lack essential information concerning the isolation procedures. Skeletal muscle was digested with collagenase I [41,42,98], collagenase type II [93], collagenase type IV [92] collagenase type XI [29,46,91], collagenase NB6 [69], trypsin [91] and and/or dispase II [44,45,91]. Intestinal smooth muscle was digested using collagenase I [94] or collagenase II [79,93,95,97,99,100,101,102,103,119]. Enteric neurons were isolated after the digestion of tissue with collagenase II and dispase II [15,95,97,102,103]. For bone-marrow MSC, bones were flushed, and bone marrow collected, washed, sometimes fractionated with density gradient and plated [18,31,32,35,36,37,38]. For fat multipotent cells, adipose tissue was digested with collagenase I [23,26,27,71].

### 3.3. Methods for Multipotent Cell Characterization

The characterization of isolated multipotent cells is mandatory for reproducibility but also for quality purpose, especially when a clinical application for ASI treatment is foreseen. Among publications using muscle multipotent cells, only 13 studies reported or referred to a proper characterization of the cells and were heterogeneous for markers (Table 3) [28,29,41,42,44,45,46,48,66,67,68,69,93]. Indeed, international criteria for MSC definition were not always applied/fulfilled/verified [120].

Cells of bone marrow origin were used in nine publications [15,18,19,22,31,33,36,40,118] and were well characterized only in seven [15,18,19,31,33,35,38]. Cells originating from adipose tissue were more often precisely characterized and were at least CD90^+^ and CD45^−^ [23,26,27,30,71]. Satellite cells have typical features such as the expression of the transcription factor PAX7 (Figure 1) [104] but there is currently a lack of standardization in the nomenclature and characterization of other myogenic cells [121]. Thus, multipotent cells from a skeletal origin were widely used to treat in vivo models of ASI, but their efficacy, as well as their identification, remained elusive. As the cell types used were insufficiently characterized, it cannot be excluded that the beneficial effect on ASI was partially mediated by co-isolated contaminant multipotent cells from connective tissue. Moreover, if we assume that satellite cells were responsible for the positive effects of skeletal muscle cell preparation on ASI, this effect was thus induced by their action on the skeletal muscle of EAS and not on the smooth muscle of IAS.

## 4. Multipotent Cell Transplantation

### 4.1. Practical Considerations

Before transplantation, cells were cultivated on plastic dishes and the number of passages before injection in the identified clinical trials ranged from three to ten. The number of injected cells, clearly reported by identified in vivo reports, ranged from 10,000 up to 90 million. In clinical trials, this number ranged from 200,000 up to 2 billion. However, the minimum number of cells required to obtain a beneficial effect on ASI remains elusive and only a few authors performed a real titration [28,92]. Thus, an excess of cells was used to compensate stem cell and progenitor cell death. Transplanted cell survival is a main concern in the field of cell therapy and it can be impaired by several factors related to the mechanical force applied during cell application, detachment from cell substrate and receiving site with inflammation and/or local hypoxia [122]. Indeed, forces generated during injection with a syringe needle are sufficient to induce up to 40% of cell death [122]. Thus, strategies have been developed to improve cell engraftment and transplantation success.

### 4.2. Adjuvant Therapy

As a strategy to limit cellular stress due to transplantation, some authors proposed to protect cells with biomaterials [44,45,46,110]. Indeed, it is known that preserving cell-extracellular substrate interactions can limit stem cell apoptosis [122]. Biomaterials were typically scaffolds of decellularized matrices or hydrogel polymers [23,33,34,35,43]. Deserving the same purpose, multipotent cells were also transplanted as sheets of cells instead of individual cells [26]. Alternatively, Trébol et al. seeded suture thread with MSC to be used for sphincter reconstruction [27] whereas Ding et al. reinforced reconstruction with a patch of an acellular dermal matrix also seeded with MSC [33].

As mentioned earlier, in some studies, authors performed immediate injection of cells along with sphincter repair, confronting cells with an acute inflammatory environment that presumably precludes cell survival [122]. On the other hand, inflammation can also enhance cell settling and homing, and the injection of myoblasts in healthy regions of EAS did not restore continence compared to the injection of cells into injured parts [28,122]. Other known strategies to improve cell survival implicated pre-conditioning of cells with either thermal preconditioning, hypoxic preconditioning, acidic preconditioning or nutrient deprivation preconditioning [122]. The goal of these strategies is to induce anti-apoptotic protein expression [122]. Injection sites were also prepared, and electrical stimulation was used in two in vivo studies [37,38] and two clinical studies to promote the homing of cells into anal sphincters [66,68]. Moreover, laser beam stimulation along with cell therapy was used in one study to promote muscle proliferation [30].

Further, growth factors (SDF-1, FGF) described to promote stem cell implantation were delivered in situ by bioscaffolds or through osmotic pumps [35,37,99,100,103]. Two studies transfected cells with SDF-1 plasmids before implantation [35,36]. As an alternative to the use of growth factors, platelet rich plasma, which is known to contain numerous growth factors, might be transplanted conjointly with stem cells [123].

### 4.3. Measure of Outcomes and Results

For in vitro studies, physiological functional evaluation was carried out in almost all identified studies to assess the contractility potential of constructed sphincters. For in vivo studies, outcomes were highly variable: The most common methods for outcome assessment were histology, anorectal manometry, physiological functionality evaluation and electromyography or electrophysiology. Some authors tracked cells using magnetic resonance imaging [42] or labeled them with fluorescent proteins (GFP) [91].

The determination of the outcome in a clinical study on anal incontinence is challenging. Different definitions of ASI exists, including incontinence to gases or not [1]. Further, in order to be comparable to the literature, authors are choosing outcomes that appear to be a gold standard in the medical literature. In almost all published clinical studies, one incontinence score was used as the primary outcome. However, a recent analysis of different incontinence scores pointed out that no single score reaches relevant psychometric soundness and recommended the use of at least two scores to evaluate ASI [124]. However, the utilization of objective outcomes may be more reliable such as high-resolution anal manometry or contact EMG (For e.g., MAPLe^®^ device, Medtronic, Dublin, Ireland [125]).

### 4.4. Results

A review of the literature identified a total of 52 original publications. Seven publications reported in vitro results (Table S2) [79,93,94,95,97,98,119], with six on bioengineered constructs among them [79,93,94,95,97,98]. One publication reported the isolation of cells from human IAS and EAS and assessed their viability [93]. In vivo experiments were reported in 38 publications (Table S1) [15,18,19,21,22,23,24,25,26,27,28,29,30,31,32,33,34,35,37,38,40,41,42,43,44,45,46,48,91,92,96,99,100,101,102,103], including five articles on heterotopic sphincter bioconstruct implantation [96,99,100,101,103]. Seven human studies were identified (Table 2) [66,67,68,69,71,72,74], including three randomized controlled trials [69,70,71,72] and one case report [74]. A total of 83 patients received cell therapy for ASI treatment.

Almost all patients included in clinical trials exhibited EAS injury. Four out of seven studies used the Wexner score as the primary outcome. The FIQL score was used in two studies. Other variables measured were anorectal manometry (5/7), endoanal ultrasonography (3/7) and electromyography (EMG) (3/7). The longest follow-up was reported by the group of Frudinger et al. who injected autologous myoblasts into EAS in 10 voluntary women with EAS defect or atrophy [66]. After a follow-up of 5 years, the mean Wexner score decreased from 15.3 (SD-2.4) before intervention to 0.7 (SD 1.3) (*p* > 0.001). In addition, anal manometry demonstrated an improvement of median resting and squeeze pressures (20 (IQR 17–28) to 32 (25–43) and 23 (IQR 20–34) to 33 (IQR 31–66), respectively). The same group started a second trial including 34 females and 5 males and found a reduction of Wexner score of −16.2 (SD-3.66) for women and -18.8 (SD-1.30) for male at one year. These results were better than sphincteroplasty, which typically induces a long-term reduction of −1 to −5.2 of median/mean Wexner score (mean follow-up between seven and eight years) [126,127]. In a similar study (muscle tissue-derived multipotent cells injected into EAS), Boyer et al. reported a reduction of median Wexner score of −6.4 (range −12 to 2) (*p* = 0.006) for the intervention group and a reduction of −1 (range −8 to 6) (*p* = 0.35) for the placebo [69]. However, using adipose tissue-derived multipotent cells in a randomized triple blinded placebo-controlled trial, De la Portilla et al. failed to demonstrate any effect on Wexner score of cell transplantation into EAS defect [72]. Globally, all seven reports described encouraging results regarding at least one of the measured outcomes (which was not necessary the primary outcome).

Until now, in vivo experiments have demonstrated a relative oncological safety of such a strategy. One in vivo study described focal cell growth at the injection site but without malignant characteristics [25]. Recent evidence confirmed that pure stem cell cultures, particularly MSC, did not develop malignant cells [128,129]. However, MSC have opposite effects on tumor cells and can promote or suppress tumor growth in vitro and in vivo [130]. Published clinical trials, which used either skeletal muscle tissue or adipose tissue multipotent cells, demonstrated that the procedures were safe.

## 5. Conclusions

Preclinical and clinical studies have demonstrated the safety of multipotent cell utilization for ASI. Published clinical studies have shown promising results, but only three of them were controlled with placebo injection [69,70,71,72]. These controlled studies had relative short follow-ups considering that ASI is a disease evolving over time. Thus, all the presented strategies for cell therapy in the context of ASI deserve further randomized controlled trials with improved methodology regarding outcome measurement and follow-up.

The ideal therapy for ASI should be cost-effective with a long-lasting effect. Apart from research and development costs, good manufacturing practices, GMP certifications and implementation charges, routine use of cell therapy appears to be highly costly [131]. Trébol et al. estimated the maximal production costs in Spain to be 7400 USD for 40 million autologous fat-derived cells or 8500 € for 100 million allogeneic fat-derived cells [130]. In our hospital, the cost for a sphincteroplasty, with a typical length of stay of two days, is 5000 USD. Recently, Gräs et al. proposed a cost-effective alternative to cell transplantation for anal sphincter regeneration. Following promising results for urinary incontinence, the authors discussed the possibility to inject fragmented muscle fibers, instead of expanded cells, into injured anal sphincters [121].

After transplantation, progenitor cells and stem cells might act by paracrine effects and/or by differentiation into functional muscular cells. It should be pointed out that the exact underlying mechanism remains poorly understood, and that basic research on this topic is still required to understand which factors and conditions are leading to cell engraftment, differentiation and finally tissue regeneration [123,132]. Moreover, the natural history of sphincter lesion/repaired sphincter healing should be better understood in order to select appropriate cell preparations and transplantation techniques. Some groups reported a different approach, considering the use of cell therapy as an add-on to sphincter repair, either directly after lesion, to simulate the primary repair of an acute obstetrical tear or at distance [18,19,20,21,22,23,24,25]. Thus, it remains to be determined how stem cells and progenitor cells should be used for ASI: As a substitution to surgery or along with surgery.

## Figures and Tables

**Table 1:** Models for anal sphincter incontinence. NHP: non-human primate.

	Designation of Model	Time Lapse between Injury and Intervention	Publication Reporting the Model	Species	Procedure	Sphincter
**Acute Anal Sphincter Injury**	Sphincterotomy and repair	0	Mazzanti et al., 2016 [18], Lorenzi et al., 2008 [19]	Rat	Sphincterotomy and primary repair of sphincters	IAS and EAS
	Repaired sphincterotomy	0	Fitzwater et al., 2015 [20], White et al 2010 [21], Pathi et al., 2012 [22]	Rat	Full thickness 7 mm incision of sphincters followed by repair	IAS and EAS
	Anal sphincter injury	0	Kuismanen et al., 2018 [23]	Rat	Incision of full thickness sphincter with mucosa followed by mucosa and IAS repair	IAS and EAS
	Proctoepisiotomy	0	Lane et al., 2013 [24], Jacobs et al., 2013 [25]	Rat	Proctoepisiotomy with repair	EAS
	Sphincterotomy	0	Inoue et al., 2018 [26]	Rat	Removal of a left semicircle of sphincter	IAS and EAS
	Extra-mucosal myotomy	0	Trébol et al., 2018 [27]	Rat	1 cm-long incision preserving the mucosa	IAS and EAS
	Anal sphincter cryoinjury	0	Bisson et al., 2013 [28]	Rat	Two cryoinjuries of sphincters at 24 h interval with liquid nitrogen on a 90° sector	IAS and EAS
	Anal sphincter cryoinjury	0	Kang et al., 2008 [29]	Rat	Cryoinjury of right hemi-sphincters	IAS and EAS
	Sphincterotomy	0	Sarveazad et al., 2019 [30]	Rabbit	Left lateral sphincterotomy	IAS and EAS
	Sphincterotomy	24 h	Salcedo et al., 2013 [15]	Rat	2–3 mm-thick transection of sphincters	IAS and EAS
	Pudendal nerve crush	24 h	Salcedo et al., 2013 [15]	Rat	Posterior incision of sacro-coccygeal area and 30 s crushing of the nerves on both sides	na
**Unrepaired Anal Sphincter Injury**	Partial anal sphincter excision	24 h and 3 weeks	Salcedo et al., 2014 [31], Li et al., 2020 [32]	Rat	Excision of 1/3 of ventral anal sphincters	IAS and EAS
	Anal sphincter injury	2 weeks	Ding et al.,2016 [33]	Rat	0.2 cm-long sphincters incision	IAS and EAS
	Unrepaired sphincterotomy	2 weeks	Montoya et al., 2015 [34]	Rat	Full thickness 7 mm incision of sphincters	IAS and EAS
	Chronic large anal sphincter defect	3 weeks	Sun et al., 2017 [35], Sun et al., 2017 [36], Sun et al., 2016 [37]	Rat	50% excision of ventral portion of anal sphincters	IAS and EAS
	Anal sphincter damage	nd	Li et al., 2018 [38]	Rat	3 mm-long incision in the right posterolateral sphincter	IAS and EAS
	Intersphincteric resection model	na	Yamaguchi et al., 2013 [39]	Rat	50% excision of IAS and a part of EAS	IAS and EAS
	Sphincterotomy	2 weeks	Aghaee-Afshar et al., 2009 [40]	Rabbit	Right lateral sphincterotomy	EAS
	Excision of external anal sphincter	3 to 24 weeks	Kajbafzadeh et al., 2016 [41], Elmi et al., 2014 [42], Kajbafzadeh et al., 2010 [43]	Rabbit	Subtotal to total excision of posterior sphincter	EAS
	Sphincter injury	4 weeks	Oh et al., 2015 [44], Oh et al., 2015 [45], Kang et al., 2013 [46]	Dog	Resection of 25% of posterior anal sphincters	IAS and EAS
	Internal sphincter hemi-sphincterectomy	6–8 weeks	Bohl et al., 2017 [47], Dadhich et al., 2019 [48]	Rabbit, NHP	50% excision of ventral portion of anal sphincter	IAS

**Table 2:** List of clinical studies on cell therapy for anal incontinence treatments. bn: billion, mio: million, FIQL: Fecal Incontinence Quality of Life.

Publication	Design	Women	Men	Concerned Sphincter	Cellular Therapy	Passages *n*	Number of Injected Cell	Outcomes	Follow-Up
De la Portilla et al. 2020 [72]	Randomized controlled trial	6	2	EAS	Autologous adipose tissue derived multipotent cells injected into EAS lesion	Up to 7	4 bn	Wexner incontinence score, FIQL, safety and feasibility, manometry	12 months
Frudinger et al. 2018 [68]	Explorative baseline controlled study	34	5	EAS	Anal electrical stimulation followed by 12 injections of autologous myoblasts from pectoralis muscle into the entire EAS.	3	25 mio	Wexner incontinence score and FIQL score, anorectal manometry	12 months
Boyer et al. 2018 [69]	Randomized controlled trial	12	0	EAS	Autologous myoblasts derived from quadriceps injected at 8 points into the EAS under endoanal ultrasonography guindance	3	0.2 to 2 bn	Wexner incontinence score	12 months
Sarvezaad et al. 2017 [71]	Randomized controlled trial	7	2	EAS	Allogenic hADSC injection along with external sphincteroplasty	5	6 mio	Endoanal ultrasonography and EMG	2 months
Romaniszyn et al. 2015 [67]	Pilot study	10	1	EAS	Autologous myoblasts injected into EAS under ultrasound guidance	7–10 passages	100 mio	Anorectal manometry, EMG, endoanal ultrasonography	12 months
Frudinger et al. 2010/2015 [73,66]	Pilot study	10	0	EAS	Anal electrical stimulation followed by 12–14 injections of autologous myoblasts from pectoralis muscle into the defect and the edges of EAS	-	20.16 mio	Wexner incontinence score, anorectal manometry (contractile tone), endoanal ultrasonography	60 months
Romaniszyn et al. 2013 [74]	Case report	0	1	IAS and EAS	Injection of autologous myoblast from quadriceps into the scar tissue of the EAS.	6–7	600 mio	Anorectal manometry, EMG	12 months

**Table 3:** Characteristics of primary cells proposed for cell therapy of anal sphincter incontinence. MHC: myosin heavy chain, Sma: α-smooth-muscle-actin, and MyoG: moygenin.

	Publication	Cells Origin	Species	Surface Antigens Expressed	Surface Antigens Not Expressed	GENE Expression	Intracellular Protein Expressed	Intracellular Protein Not Expressed	Differentiation Test
**Muscle-Derived Cells**	Bisson et al., 2015 [28]	Skeletal muscle	Rat	CD56	-	*DES, MYOD1, MYF5,*	-	-	-
	Lane et al., 2013 [24], Jacobs et al., 2013 [25], Craig et al., 2010 [92]	Skeletal muscle	Rat	-	-	-	-	-	-
	Saihara et al., 2009 [91]	Skeletal muscle	Rat	-	-	-	-	-	Myotubes
	Kang et al., 2008 [29]	Skeletal muscle	Rat	CD34	CD45	-	Desmin	-	-
	Kajbafzadeh et al., 2016 [41]	Skeletal muscle	Rabbit	-	-	-	Pax7, Desmin	-	Myotubes
	Elmi et al., 2014 [42]	Skeletal muscle	Rabbit	-	-	-	Desmin, MyoD	-	
	Oh et al., 2015 [44], Oh et al., 2015 [45]	Skeletal muscle	Dog	-	-	-	Pax7, Sma	MHC, MyoG	Myotubes
	Kang et al., 2013 [46]	Skeletal muscle	Dog	-	-	*-*	Pax7	MHC	α-SMA
	Boyer et al., 2018 [69]	Skeletal muscle	Human	CD90, HLA-I	CD34, CD45, CD133	*DES, MYOD1, MYF5, PAX7*	-	-	-
	Frudinger et al., 2015 [66], Frudinger et al., 2018 [68]	Skeletal muscle	Human	SSEA3, SSEA4, CD56, CD90	-	*NANOG1, NACAM1, MYOD1, PAX7, PAX3, MYF5, DES, MYOG*	Desmin, UTF1, Pax7, Myf5	-	Myotubes
	Romaniszyn et al., 2015 [67]	Skeletal muscle	Human	CD56	-	*DES, MYOD1, MYOG*	-	-	Myocyte
	Romaniszyn et al., 2013 [74]	Skeletal muscle	Human	-	-	-	-	-	-
	Son et al., 2019 [93]	EAS	Human	CD34, NG2	-	-	Pax7	-	MyoG, MyHC
	Bohl et al., 2017 [102], Rego et al., 2017 [98]	Smooth muscle	Rabbit						
	Raghavan et al., 2010 [99], Hashish et al., 2010 [100], Miyasaka et al., 2011 [101]	IAS	Mouse	-	-	-	-	-	-
	Zakhem et al., 2015 [97], Rego et al. 2017 [98]	IAS	Rabbit	-	-	-	-	-	-
	Dadhich et al., 2019 [48]	IAS	NHP	-	-	*SMTN*	-	Sma, and smoothelin	-
	Gilmont et al., 2014 [95]	IAS	Human	-	-	-	-	-	-
	Singh and Rattan 2012 [94]	IAS	Human	-	-	-	-	-	-
	Raghavan et al., 2014 [96], Somara et al., 2009 [79]	IAS	Human	-	-	-	-	-	-
**Bone Marrow-Derived Cells**	Li et al., 2018 [38]	Bone marrow	Rat	-	CD34, CD45	-	-	-	-
	Ding et al., 2016 [33]	Bone marrow, transfected with galectin-1	Rat	CD90	CD45	-	-	-	-
	Sun et al., 2017 [36]	Bone marrow	Rat	-	-	-	-	-	-
	Mazzanti et al., 2016 [18], Lorenzi et al., 2008 [19]	Bone marrow	Rat	CD44, CD54, CD73, CD90, CD106	CD11b, CD11c, CD45	-	-	-	Osteogenic and adipogenic
	Salcedo et al., 2014 [31], Salcedo et al., 2013 [15]	Bone marrow	Rat	-	CD34, CD45	-	-	-	-
	Pathi et al., 2012 [22]	Bone marrow	Rat	-	-	-	-	-	-
	Aghaee-Afshar et al., 2009 [40]	Bone marrow	Rabbit	-	-	-	-	-	-
**Adipose Tissue-Derived Cells**	Trébol et al., 2018 [27]	Adipose tissue	Rat	CD29, CD90	CD11n, CD45	-	-	-	-
	Inoue et al., 2018 [26]	Adipose tissue	Rat	CD90	CD31, CD45	-	-	-	Adipogenic and myogenic
	Sarveazad et al., 2019 [30]	Adipose tissue	Human	CD29, CD73, CD105	CD34, CD45				
	Sarveazad et al., 2017 [71]	Adipose tissue	Human	CD44, CD73, CD90	CD31, CD45	-	-	-	-
	Kuismanen et al., 2018 [23]	Adipose tissue	Human	CD73, CD90, CD105	CD14, CD19, CD34, CD45RO, CD54, HLA-DR	-	-	-	-
**Neural Tissue-Derived Cells**	Bohl et al., 2017 [102]	Enteric Neural System	Rabbit	-	-	-	-	-	-
	Zakhem et al., 2015 [97]	Appendix neuronal system	Rabbit	P75(NTR)	-	-	Sox2, Nestin		Neurospheres
	Rego et al., 2017 [98]	Enteric neuronal system	Rabbit	-	-	-	-	-	Neurospheres
	Dadhich et al., 2019 [48]	Enteric neuronal system	NHP	P75(NTR)				smoothelin, oct4	
	Gilmont et al., 2014 [95]	Enteric neuronal system	Human	P75(NTR)	-	-	-	-	Neurospheres
	Raghavan et al., 2014 [96], Raghavan et al., 2011 [103]	Enteric neuronal system	Human	-	-	-	-	-	-
**Miscellaneous**	Aghaee-Afshar et al., 2009 [40]	Umbilical cord matrix	Human	-	-	-	-	-	-

## Supplementary Materials

The following are available online at https://www.mdpi.com/2073-4409/10/8/2086/s1, Table S1. List and description of identified in vivo publications investigating cell therapy for the treatment of anal sphincter incontinence, Table S2. List of in vitro publications.
