# Correction: Balaphas et al. Cell Therapy for Anal Sphincter Incontinence: Where Do We Stand? *Cells* 2021, *10*, 2086

**DOI:** 10.3390/cells12242857

**Published:** 2023-12-18

**Authors:** Alexandre Balaphas, Jeremy Meyer, Raphael P. H. Meier, Emilie Liot, Nicolas C. Buchs, Bruno Roche, Christian Toso, Leo H. Bühler, Carmen Gonelle-Gispert, Frédéric Ris

**Affiliations:** 1 Division of Digestive Surgery, University Hospitals of Geneva, 1205 Geneva, Switzerland; jeremy.meyer@hcuge.ch (J.M.); emilie.liot@hcuge.ch (E.L.); nicolas.c.buchs@hcuge.ch (N.C.B.); bruno.roche@grangettes.ch (B.R.); christian.toso@hcuge.ch (C.T.); frederic.ris@hcuge.ch (F.R.); 2 Department of Surgery, Geneva Medical School, University of Geneva, 1205 Geneva, Switzerland; 3 Department of Surgery, University of Maryland School of Medicine, Baltimore, MD 21201, USA; rmeier@som.umaryland.edu; 4 Faculty of Science and Medicine, University of Fribourg, 1700 Fribourg, Switzerland; leo.buhler@unifr.ch (L.H.B.); carmen.gonelle@unifr.ch (C.G.-G.)

The authors would like to add a new reference to the section “*3.1. Multipotent Cell Origins*”, which was missing in the original version [1].

In the original publication, Ref. [111] was not cited correctly, it should change as follows:

New Ref. 111: Sanabria-de la Torre, R.; Quiñones-Vico, M.I.; Fernández-González, A.; Sánchez-Díaz, M.; Montero-Vílchez, T.; Sierra-Sánchez, Á.; Arias-Santiago, S. Alloreactive Immune Response Associated to Human Mesenchymal Stromal Cells Treatment: A Systematic Review. *J. Clin. Med.* **2021**, *10*, 2991.

The citation has now been inserted in *Subsection 3.1. “Multipotent Cell origins”*, Paragraph number 5 and should read:

“However, recent evidence demonstrated the development of donor-specific antibodies, and MSC rejection has been documented [111].”

In the original publication, many citations and reference numbers were shifted. Several reference citations did not correspond to the reference numbers next to them. Corrections were made in Tables 1 and 3 as well as in several Sections and Subsections.

In Table 1, we would like to update the references in Column number 4. Thus, Table 1 will be updated from:

**Table 1:** Models for anal sphincter incontinence; NHP: non-human primate.

	Designation of Model	Time Lapse between Injury and Intervention	Publication Reporting the Model	Species	Procedure	Sphincter
**Acute Anal Sphincter Injury**	Sphincterotomy and repair	0	Mazzanti et al., 2016 [27], Lorenzi et al., 2008 [26]	Rat	Sphincterotomy and primary repair of sphincters	IAS and EAS
	Repaired sphincterotomy	0	Fitzwater et al., 2015 [28], White et al., 2010 [29], Pathi et al., 2012 [30]	Rat	Full thickness 7 mm incision of sphincters followed by repair	IAS and EAS
	Anal sphincter injury	0	Kuismanen et al., 2018 [31]	Rat	Incision of full thickness sphincter with mucosa followed by mucosa and IAS repair	IAS and EAS
	Proctoepisiotomy	0	Lane et al., 2013 [32], Jacobs et al., 2013 [33]	Rat	Proctoepisiotomy with repair	EAS
	Sphincterotomy	0	Inoue et al., 2018 [34]	Rat	Removal of a left semicircle of sphincter	IAS and EAS
	Extra-mucosal myotomy	0	Trébol et al., 2018 [35]	Rat	1 cm long incision preserving the mucosa	IAS and EAS
	Anal sphincter cryoinjury	0	Bisson et al., 2013 [36]	Rat	Two cryoinjuries of sphincters at 24 h interval with liquid nitrogen on a 90° sector	IAS and EAS
	Anal sphincter cryoinjury	0	Kang et al., 2008 [37]	Rat	Cryoinjury of right hemi-sphincters	IAS and EAS
	Sphincterotomy	0	Sarveazad et al., 2019 [38]	Rabbit	Left lateral sphincterotomy	IAS and EAS
	Sphincterotomy	24 h	Salcedo et al., 2013 [15]	Rat	2–3-mm thick transection of sphincters	IAS and EAS
	Pudendal nerve crush	24 h	Salcedo et al., 2013 [15]	Rat	Posterior incision of sacro-coccygeal area and 30 s crushing of the nerves on both sides	na
**Unrepaired Anal Sphincter Injury**	Partial anal sphincter excision	24 h and 3 weeks	Salcedo et al., 2014 [39], Li et al., 2020 [40]	Rat	Excision of 1/3 of ventral anal sphincters	IAS and EAS
	Anal sphincter injury	2 weeks	Ding et al.,2016 [41]	Rat	0.2 cm long sphincters incision	IAS and EAS
	Unrepaired sphincterotomy	2 weeks	Montoya et al., 2015 [42]	Rat	Full thickness 7 mm incision of sphincters	IAS and EAS
	Chronic large anal sphincter defect	3 weeks	Sun et al., 2017 [43], Sun et al., 2017 [44], Sun et al., 2016 [45]	Rat	50% excision of ventral portion of anal sphincters	IAS and EAS
	Anal sphincter damage	nd	Li et al., 2018 [46]	Rat	3 mm long incision in the right posterolateral sphincter	IAS and EAS
	Intersphincteric resection model	na	Yamaguchi et al., 2013 [47]	Rat	50% excision of IAS and a part of EAS	IAS and EAS
	Sphincterotomy	2 weeks	Aghaee-Afshar et al., 2009 [48]	Rabbit	Right lateral sphincterotomy	EAS
	Excision of external anal sphincter	3 to 24 weeks	Kajbafzadeh et al., 2016 [49], Elmi et al., 2014 [50], Kajbafzadeh et al., 2010 [51]	Rabbit	Subtotal to total excision of posterior sphincter	EAS
	Sphincter injury	4 weeks	Oh et al., 2015 [52], Oh et al., 2015 [53], Kang et al., 2013 [54]	Dog	Resection of 25% of posterior anal sphincters	IAS and EAS
	Internal sphincter hemi-sphincterectomy	6–8 weeks	Bohl et al., 2017 [55], Dadhich et al., 2019 [56]	Rabbit, NHP	50% excision of ventral portion of anal sphincter	IAS

To:

**Table 1:** Models for anal sphincter incontinence. NHP: non-human primate.

	Designation of Model	Time Lapse between Injury and Intervention	Publication Reporting the Model	Species	Procedure	Sphincter
**Acute Anal Sphincter Injury**	Sphincterotomy and repair	0	Mazzanti et al., 2016 [18], Lorenzi et al., 2008 [19]	Rat	Sphincterotomy and primary repair of sphincters	IAS and EAS
	Repaired sphincterotomy	0	Fitzwater et al., 2015 [20], White et al 2010 [21], Pathi et al., 2012 [22]	Rat	Full thickness 7 mm incision of sphincters followed by repair	IAS and EAS
	Anal sphincter injury	0	Kuismanen et al., 2018 [23]	Rat	Incision of full thickness sphincter with mucosa followed by mucosa and IAS repair	IAS and EAS
	Proctoepisiotomy	0	Lane et al., 2013 [24], Jacobs et al., 2013 [25]	Rat	Proctoepisiotomy with repair	EAS
	Sphincterotomy	0	Inoue et al., 2018 [26]	Rat	Removal of a left semicircle of sphincter	IAS and EAS
	Extra-mucosal myotomy	0	Trébol et al., 2018 [27]	Rat	1 cm-long incision preserving the mucosa	IAS and EAS
	Anal sphincter cryoinjury	0	Bisson et al., 2013 [28]	Rat	Two cryoinjuries of sphincters at 24 h interval with liquid nitrogen on a 90° sector	IAS and EAS
	Anal sphincter cryoinjury	0	Kang et al., 2008 [29]	Rat	Cryoinjury of right hemi-sphincters	IAS and EAS
	Sphincterotomy	0	Sarveazad et al., 2019 [30]	Rabbit	Left lateral sphincterotomy	IAS and EAS
	Sphincterotomy	24 h	Salcedo et al., 2013 [15]	Rat	2–3 mm-thick transection of sphincters	IAS and EAS
	Pudendal nerve crush	24 h	Salcedo et al., 2013 [15]	Rat	Posterior incision of sacro-coccygeal area and 30 s crushing of the nerves on both sides	na
**Unrepaired Anal Sphincter Injury**	Partial anal sphincter excision	24 h and 3 weeks	Salcedo et al., 2014 [31], Li et al., 2020 [32]	Rat	Excision of 1/3 of ventral anal sphincters	IAS and EAS
	Anal sphincter injury	2 weeks	Ding et al.,2016 [33]	Rat	0.2 cm-long sphincters incision	IAS and EAS
	Unrepairedsphincterotomy	2 weeks	Montoya et al., 2015 [34]	Rat	Full thickness 7 mm incision of sphincters	IAS and EAS
	Chronic large anal sphincter defect	3 weeks	Sun et al., 2017 [35], Sun et al., 2017 [36], Sun et al., 2016 [37]	Rat	50% excision of ventral portion of anal sphincters	IAS and EAS
	Anal sphincter damage	nd	Li et al., 2018 [38]	Rat	3 mm-long incision in the right posterolateral sphincter	IAS and EAS
	Intersphincteric resection model	na	Yamaguchi et al., 2013 [39]	Rat	50% excision of IAS and a part of EAS	IAS and EAS
	Sphincterotomy	2 weeks	Aghaee-Afshar et al., 2009 [40]	Rabbit	Right lateral sphincterotomy	EAS
	Excision of external anal sphincter	3 to 24 weeks	Kajbafzadeh et al., 2016 [41], Elmi et al., 2014 [42], Kajbafzadeh et al., 2010 [43]	Rabbit	Subtotal to total excision of posterior sphincter	EAS
	Sphincter injury	4 weeks	Oh et al., 2015 [44], Oh et al., 2015 [45], Kang et al., 2013 [46]	Dog	Resection of 25% of posterior anal sphincters	IAS and EAS
	Internal sphincter hemi-sphincterectomy	6–8 weeks	Bohl et al., 2017 [47], Dadhich et al., 2019 [48]	Rabbit, NHP	50% excision of ventral portion of anal sphincter	IAS

In Table 3, we would like to update the references in Column number 2. Thus, Table 3 will be updated from:

**Table 3:** Characteristics of primary cells proposed for cell therapy of anal sphincter incontinence. MHC: Myosin heavy chain, Sma: α-Smooth-muscle-actin, MyoG: Moygenin.

	Publication	Cells Origin	Species	Surface Antigens Expressed	Surface Antigens Not Expressed	GENE Expression	Intracellular Protein Expressed	Intracellular Protein Not Expressed	Differentiation Test
**Muscle-Derived Cells**	Bisson et al., 2015 [36]	Skeletal muscle	Rat	CD56	-	*DES*, *MYOD1*, *MYF5*,	-	-	-
	Lane et al. 2013 [32], Jacobs et al., 2013 [33], Craig et al., 2010 [92]	Skeletal muscle	Rat	-	-	-	-	-	-
	Saihara et al., 2009 [91]	Skeletal muscle	Rat	-	-	-	-	-	Myotubes
	Kang et al., 2008 [37]	Skeletal muscle	Rat	CD34	CD45	-	Desmin	-	-
	Kajbafzadeh et al., 2016 [49]	Skeletal muscle	Rabbit	-	-	-	Pax7, Desmin	-	Myotubes
	Elmi et al., 2014 [50]	Skeletal muscle	Rabbit	-	-	-	Desmin, MyoD	-	
	Oh et al., 2015 [52], Oh et al., 2015 [53]	Skeletal muscle	Dog	-	-	-	Pax7, Sma	MHC, MyoG	Myotubes
	Kang et al., 2013 [54]	Skeletal muscle	Dog	-	-	*-*	Pax7	MHC	α-SMA
	Boyer et al., 2018 [69]	Skeletal muscle	Human	CD90, HLA-I	CD34, CD45, CD133	*DES*, *MYOD1*, *MYF5*, *PAX7*	-	-	-
	Frudinger et al., 2015 [66], Frudinger et al., 2018 [68]	Skeletal muscle	Human	SSEA3, SSEA4, CD56, CD90	-	*NANOG1*, *NACAM1*, *MYOD1*, *PAX7*, *PAX3*, *MYF5*, *DES*, *MYOG*	Desmin, UTF1, Pax7, Myf5	-	Myotubes
	Romaniszyn et al., 2015 [67]	Skeletal muscle	Human	CD56	-	*DES*, *MYOD1*, *MYOG*	-	-	Myocyte
	Romaniszyn et al., 2013 [74]	Skeletal muscle	Human	-	-	-	-	-	-
	Son et al., 2019 [93]	EAS	Human	CD34, NG2	-	-	Pax7	-	MyoG, MyHC
	Bohl et al., 2017 [102], Rego et al., 2017 [98]	Smooth muscle	Rabbit						
	Raghavan et al., 2010 [99], Hashish et al., 2010 [100], Miyasaka et al., 2011 [101]	IAS	Mouse	-	-	-	-	-	-
	Zakhem et al., 2015 [97], Rego et al., 2017 [98]	IAS	Rabbit	-	-	-	-	-	-
	Dadhich et al., 2019 [56]	IAS	NHP	-	-	*SMTN*	-	Sma, and smoothelin	-
	Gilmont et al., 2014 [95]	IAS	Human	-	-	-	-	-	-
	Singh and Rattan 2012 [94]	IAS	Human	-	-	-	-	-	-
	Raghavan et al., 2014 [96], Somara et al., 2009 [79]	IAS	Human	-	-	-	-	-	-
**Bone Marrow-Derived Cells**	Li et al., 2018 [46]	Bone marrow	Rat	-	CD34, CD45	-	-	-	-
	Ding et al., 2016 [41]	Bone marrow, transfected with galectin-1	Rat	CD90	CD45	-	-	-	-
	Sun et al., 2017 [44]	Bone marrow	Rat	-	-	-	-	-	-
	Mazzanti et al., 2016 [27], Lorenzi et al., 2008 [26]	Bone marrow	Rat	CD44, CD54, CD73, CD90, CD106	CD11b, CD11c, CD45	-	-	-	Osteogenic and adipogenic
	Salcedo et al., 2014 [39], Salcedo et al., 2013 [15]	Bone marrow	Rat	-	CD34, CD45	-	-	-	-
	Pathi et al., 2012 [30]	Bone marrow	Rat	-	-	-	-	-	-
	Aghaee-Afshar et al., 2009 [48]	Bone marrow	Rabbit	-	-	-	-	-	-
**Adipose Tissue-Derived Cells**	Trébol et al., 2018 [35]	Adipose tissue	Rat	CD29, CD90	CD11n, CD45	-	-	-	-
	Inoue et al., 2018 [34]	Adipose tissue	Rat	CD90	CD31, CD45	-	-	-	Adipogenic and myogenic
	Sarveazad et al., 2019 [38]	Adipose tissue	Human	CD29, CD73, CD105	CD34, CD45				
	Sarveazad et al., 2017 [71]	Adipose tissue	Human	CD44, CD73, CD90	CD31, CD45	-	-	-	-
	Kuismanen et al., 2018 [31]	Adipose tissue	Human	CD73, CD90, CD105	CD14, CD19, CD34, CD45RO, CD54, HLA-DR	-	-	-	-
**Neural Tissue-Derived Cells**	Bohl et al., 2017 [102]	Enteric Neural System	Rabbit	-	-	-	-	-	-
	Zakhem et al., 2015 [97]	Appendix neuronal system	Rabbit	P75(NTR)	-	-	Sox2, Nestin		Neurospheres
	Rego et al., 2017 [97]	Enteric neuronal system	Rabbit	-	-	-	-	-	Neurospheres
	Dadhich et al., 2019 [56]	Enteric neuronal system	NHP	P75(NTR)				smoothelin, oct4	
	Gilmont et al., 2014 [95]	Enteric neuronal system	Human	P75(NTR)	-	-	-	-	Neurospheres
	Raghavan et al., 2014 [96], Raghavan et al., 2011 [103]	Enteric neuronal system	Human	-	-	-	-	-	-
**Miscellaneous**	Aghaee-Afshar et al., 2009 [48]	Umbilical cord matrix	Human	-	-	-	-	-	-

To:

**Table 3:** Characteristics of primary cells proposed for cell therapy of anal sphincter incontinence. MHC: myosin heavy chain, Sma: α-smooth-muscle-actin, and MyoG: moygenin.

	Publication	Cells Origin	Species	Surface Antigens Expressed	Surface Antigens Not Expressed	GENE Expression	Intracellular Protein Expressed	Intracellular Protein Not Expressed	Differentiation Test
**Muscle-Derived Cells**	Bisson et al., 2015 [28]	Skeletal muscle	Rat	CD56	-	*DES*, *MYOD1*, *MYF5*,	-	-	-
	Lane et al., 2013 [24], Jacobs et al., 2013 [25], Craig et al., 2010 [92]	Skeletal muscle	Rat	-	-	-	-	-	-
	Saihara et al., 2009 [91]	Skeletal muscle	Rat	-	-	-	-	-	Myotubes
	Kang et al., 2008 [29]	Skeletal muscle	Rat	CD34	CD45	-	Desmin	-	-
	Kajbafzadeh et al., 2016 [41]	Skeletal muscle	Rabbit	-	-	-	Pax7, Desmin	-	Myotubes
	Elmi et al., 2014 [42]	Skeletal muscle	Rabbit	-	-	-	Desmin, MyoD	-	
	Oh et al., 2015 [44], Oh et al., 2015 [45]	Skeletal muscle	Dog	-	-	-	Pax7, Sma	MHC, MyoG	Myotubes
	Kang et al., 2013 [46]	Skeletal muscle	Dog	-	-	*-*	Pax7	MHC	α-SMA
	Boyer et al., 2018 [69]	Skeletal muscle	Human	CD90, HLA-I	CD34, CD45, CD133	*DES*, *MYOD1*, *MYF5*, *PAX7*	-	-	-
	Frudinger et al., 2015 [66], Frudinger et al., 2018 [68]	Skeletal muscle	Human	SSEA3, SSEA4, CD56, CD90	-	*NANOG1*, *NACAM1*, *MYOD1*, *PAX7*, *PAX3*, *MYF5*, *DES*, *MYOG*	Desmin, UTF1, Pax7, Myf5	-	Myotubes
	Romaniszyn et al., 2015 [67]	Skeletal muscle	Human	CD56	-	*DES*, *MYOD1*, *MYOG*	-	-	Myocyte
	Romaniszyn et al., 2013 [74]	Skeletal muscle	Human	-	-	-	-	-	-
	Son et al., 2019 [93]	EAS	Human	CD34, NG2	-	-	Pax7	-	MyoG, MyHC
	Bohl et al., 2017 [102], Rego et al., 2017 [98]	Smooth muscle	Rabbit						
	Raghavan et al., 2010 [99], Hashish et al., 2010 [100], Miyasaka et al., 2011 [101]	IAS	Mouse	-	-	-	-	-	-
	Zakhem et al., 2015 [97], Rego et al. 2017 [98]	IAS	Rabbit	-	-	-	-	-	-
	Dadhich et al., 2019 [48]	IAS	NHP	-	-	*SMTN*	-	Sma, and smoothelin	-
	Gilmont et al., 2014 [95]	IAS	Human	-	-	-	-	-	-
	Singh and Rattan 2012 [94]	IAS	Human	-	-	-	-	-	-
	Raghavan et al., 2014 [96], Somara et al., 2009 [79]	IAS	Human	-	-	-	-	-	-
**Bone Marrow-Derived Cells**	Li et al., 2018 [38]	Bone marrow	Rat	-	CD34, CD45	-	-	-	-
	Ding et al., 2016 [33]	Bone marrow, transfected with galectin-1	Rat	CD90	CD45	-	-	-	-
	Sun et al., 2017 [36]	Bone marrow	Rat	-	-	-	-	-	-
	Mazzanti et al., 2016 [18], Lorenzi et al., 2008 [19]	Bone marrow	Rat	CD44, CD54, CD73, CD90, CD106	CD11b, CD11c, CD45	-	-	-	Osteogenic and adipogenic
	Salcedo et al., 2014 [31], Salcedo et al., 2013 [15]	Bone marrow	Rat	-	CD34, CD45	-	-	-	-
	Pathi et al., 2012 [22]	Bone marrow	Rat	-	-	-	-	-	-
	Aghaee-Afshar et al., 2009 [40]	Bone marrow	Rabbit	-	-	-	-	-	-
**Adipose Tissue-Derived Cells**	Trébol et al., 2018 [27]	Adipose tissue	Rat	CD29, CD90	CD11n, CD45	-	-	-	-
	Inoue et al., 2018 [26]	Adipose tissue	Rat	CD90	CD31, CD45	-	-	-	Adipogenic and myogenic
	Sarveazad et al., 2019 [30]	Adipose tissue	Human	CD29, CD73, CD105	CD34, CD45				
	Sarveazad et al., 2017 [71]	Adipose tissue	Human	CD44, CD73, CD90	CD31, CD45	-	-	-	-
	Kuismanen et al., 2018 [23]	Adipose tissue	Human	CD73, CD90, CD105	CD14, CD19, CD34, CD45RO, CD54, HLA-DR	-	-	-	-
**Neural Tissue-Derived Cells**	Bohl et al., 2017 [102]	Enteric Neural System	Rabbit	-	-	-	-	-	-
	Zakhem et al., 2015 [97]	Appendix neuronal system	Rabbit	P75(NTR)	-	-	Sox2, Nestin		Neurospheres
	Rego et al., 2017 [98]	Enteric neuronal system	Rabbit	-	-	-	-	-	Neurospheres
	Dadhich et al., 2019 [48]	Enteric neuronal system	NHP	P75(NTR)				smoothelin, oct4	
	Gilmont et al., 2014 [95]	Enteric neuronal system	Human	P75(NTR)	-	-	-	-	Neurospheres
	Raghavan et al., 2014 [96], Raghavan et al., 2011 [103]	Enteric neuronal system	Human	-	-	-	-	-	-
**Miscellaneous**	Aghaee-Afshar et al., 2009 [40]	Umbilical cord matrix	Human	-	-	-	-	-	-

References were updated in the following sections and subsections:

In *Subsection 2.2. Acute Anal Sphincter Injury and Healing*, all the references were updated from:

The classical clinical situation of acute anal sphincter injury is well illustrated by childbirth trauma, which still occurs in 11% of vaginal deliveries and can extend up to the IAS and sometimes the rectum (starting from posterior wall of vagina) [18] (Figure 1). In the case of extending traumatism to the level of rectal wall, dedicated stem cells of the anal canal transition zone, positive for cytokeratin 17, participate in the healing process of the mucosa of the rectum and the anal canal [19]. On the other hand, the healing process of anal sphincters has not been thoroughly studied and it is supposed to be very similar to other muscle-healing processes [20]. Under optimal conditions, healing ultimately leads to the generation of new myofibers/smooth muscle cells from muscle satellite cells/progenitor smooth muscle cells or the reparation of damaged myofibers after fusion with muscle satellite cells [21,22]. After anal sphincter injury, it is thought that inflammatory cells’ cross-talk produces cytokines and growth factors that will recruit stem cells and progenitor cells [20,22]. These cells could be mobilized from bone marrow or the surrounding tissues and might favor healing, be incorporated into the wound or further recruit stem cells and progenitor cells [20,22,23]. Especially, skeletal muscle satellite cells, which are localized in periphery of myofibers, near vascular or nerve structures, are a source of myoblasts and, further, new myocytes after their activation [24,25]. However, Lorenzi et al., reported in rats, after direct anal sphincter injury and repair, the persistence of fibrous tissue with dilated blood vessels and muscle cell degeneration patterns [26].

Progenitor cells produce cytokines like stromal-derived factor 1 (SDF-1) that are both chemoattractive for progenitor cells (including myoblasts and smooth muscle progenitor cells) but also contribute to cell proliferation, migration and survival [20,57,58]. SDF-1 seems to be a prominent cytokine for anal sphincter healing. Salcedo et al., reported a rapid local burst of SDF-1 and monocyte chemotactic protein-3 (MCP-3) expression in rats one hour after sphincter injury and up to 21 days after injury [59]. Moreover, an injection of plasmids with SDF-1 directly into EAS muscle or transplantation of SDF-1 transfected progenitor cells both improved continence in rodents after partial sphincterectomy with the same extend [43,44].

In theory, progenitor cells could be transplanted soon after injury and this is considered to be the best option to maximize the effect of transplantation (Figure 2) [20]. Indeed, it is supposed that progenitor cells might increase the natural healing process notably through the local release of cytokines such as SDF-1 [20,59]. This strategy was evaluated in several preclinical studies where progenitor cells were either directly injected after injury with surgical reparation, or not, to mimic the situation of a direct repair of sphincters [26–33,40]. Of note, some authors delayed this intervention by 24 h [15,39,40,46]. To the best of our knowledge, progenitor cells have never been injected in a patient with acute anal sphincter injury.

To:

The classical clinical situation of acute anal sphincter injury is well illustrated by childbirth trauma, which still occurs in 11% of vaginal deliveries and can extend up to the IAS and sometimes the rectum (starting from posterior wall of vagina) [49] (Figure 1). In the case of extending traumatism to the level of rectal wall, dedicated stem cells of the anal canal transition zone, positive for cytokeratin 17, participate in the healing process of the mucosa of the rectum and the anal canal [50]. On the other hand, the healing process of anal sphincters has not been thoroughly studied and it is supposed to be very similar to other muscle-healing processes [51]. Under optimal conditions, healing ultimately leads to the generation of new myofibers/smooth muscle cells from muscle satellite cells/progenitor smooth muscle cells or the reparation of damaged myofibers after fusion with muscle satellite cells [52,53]. After anal sphincter injury, it is thought that inflammatory cells’ cross-talk produces cytokines and growth factors that will recruit stem cells and progenitor cells [51,53]. These cells could be mobilized from bone marrow or the surrounding tissues and might favor healing, be incorporated into the wound or further recruit stem cells and progenitor cells [51,53,54]. Especially, skeletal muscle satellite cells, which are localized in periphery of myofibers, near vascular or nerve structures, are a source of myoblasts and, further, new myocytes after their activation [55,56]. However, Lorenzi et al. reported in rats, after direct anal sphincter injury and repair, the persistence of fibrous tissue with dilated blood vessels and muscle cell degeneration patterns [19].

Progenitor cells produce cytokines like stromal-derived factor 1 (SDF-1) that are both chemoattractive for progenitor cells (including myoblasts and smooth muscle progenitor cells) but also contribute to cell proliferation, migration and survival [51,57,58]. SDF-1 seems to be a prominent cytokine for anal sphincter healing. Salcedo et al. reported a rapid local burst of SDF-1 and monocyte chemotactic protein-3 (MCP-3) expression in rats one hour after sphincter injury and up to 21 days after injury [59]. Moreover, an injection of plasmids with SDF-1 directly into EAS muscle or transplantation of SDF-1 transfected progenitor cells both improved continence in rodents after partial sphincterectomy with the same extend [35,36]. 

In theory, progenitor cells could be transplanted soon after injury and this is considered to be the best option to maximize the effect of transplantation (Figure 2) [51]. Indeed, it is supposed that progenitor cells might increase the natural healing process notably through the local release of cytokines such as SDF-1 [51,59]. This strategy was evaluated in several preclinical studies where progenitor cells were either directly injected after injury with surgical reparation, or not, to mimic the situation of a direct repair of sphincters [18–25,32]. Of note, some authors delayed this intervention by 24 h [15,31,32,38]. To the best of our knowledge, progenitor cells have never been injected in a patient with acute anal sphincter injury.

In *Subsection 2.3. Unrepaired Anal Sphincter Injury*, in Paragraph 1, 2 and 4, all the references were updated from:

Most often, an anal sphincter tear is usually identified after delivery and surgically repaired [18]. Sometimes, the diagnosis can be missed, resulting in an occult anal sphincter injury [60]. As for an acute anal injury, little is known about the long-term healing and remodeling of damaged muscle, and an analogy can be made with the repair processes of skeletal and smooth muscles from other localizations (cf. Section 2.2). Probably, some unrepaired anal sphincter lesions could spontaneously heal. There exists evidence that a clear cut through anal sphincters in rats can heal spontaneously without inducing ASI [39,47]. As a result, almost all models of ASI in rats imply the partial resection of anal sphincters. In rat models where simple anal sphincters section were performed, acute and chronic inflammation was seen at the site of injury, characterized by neutrophils, monocytes macrophages infiltrations and fibrous tissue [28,30], disorganization of striated fibers of the EAS [43,45], but also mucin pool inclusions with histiocytes [42]. In rabbits, Rajasekaran investigated the effects of an EAS clear-cut section over time. The authors reported early collagen deposits from one week after injury, but also extensive fibrosis appearing three weeks after injury, at the site of myotomy but also beyond [61]. The presence of fibrosis was confirmed by other authors in rabbits [48,51]. In dogs, three weeks after IAS and EAS partial excision, Kang et al. reported focal interstitial inflammation, fibrosis and atrophy of smooth and striated muscles [54].

Anal sphincters encircle the anal canal, and the loss of this circular shape, as a result of injury, directly impairs the continence function [62]. However, during sphincter repair surgery, one can observe that the retracted muscle edges are held together with fibrous tissue, which bridges the defect [63]. Thus, unrepaired damage to IAS or EAS does not evolve into a hole in the sphincter ring (EAS and/or IAS), but rather into altered tissue, which fills the breach. In rats, this tissue contained mast cells and other inflammatory cells [27]. To the best of our knowledge, the importance of this tissue was never investigated in humans but could be of importance, especially if the muscle gap is the target of cell therapy.

Another strategy to treat patients with ASI is the transplantation of a biosphincter. Bitar’s group, from the Wake Forest Institute for Regenerative Medicine, has been working for decades on the implantation of bioengineered anal sphincters composed of innervated smooth muscle cells (Table S2). The results are promising and the group recently implanted the construct in large animals, including non-human primates [55,56]. This strategy is interesting as it provides a complete and functional IAS substitute and might be part of a reconstruction strategy for patients in whom IAS has been totally or partially removed such as for ultra-low rectal cancer resections. However, IAS engineering has some hurdles to overcome. First, this approach uses IAS or digestive smooth muscle cells and intestinal neuronal cells. For translation to the clinic, tissue might be procured from organ donors, requiring further immunosuppression and exposing patients to its associated risks. Those risks need to be balanced with the fact that ASI is a non-life-threatening condition. Moreover, Araki et al., recently demonstrated the feasibility of anorectal transplantation in a dog model, with this approach being serious concurrent to allogenic biosphincters [70]. Further, the tissue culture of such constructs requires 6 to 8 weeks of culture, which increases the risk of microbial contamination. Finally, the cost of the creation of an organic construct seems to be higher than cell isolation and expansion.

To:

Most often, an anal sphincter tear is usually identified after delivery and surgically repaired [49]. Sometimes, the diagnosis can be missed, resulting in an occult anal sphincter injury [60]. As for an acute anal injury, little is known about the long-term healing and remodeling of damaged muscle, and an analogy can be made with the repair processes of skeletal and smooth muscles from other localizations (cf. Section 2.2). Probably, some unrepaired anal sphincter lesions could spontaneously heal. There exists evidence that a clear cut through anal sphincters in rats can heal spontaneously without inducing ASI [31,39]. As a result, almost all models of ASI in rats imply the partial resection of anal sphincters. In rat models where simple anal sphincters section were performed, acute and chronic inflammation was seen at the site of injury, characterized by neutrophils, monocytes macrophages infiltrations and fibrous tissue [20,22], disorganization of striated fibers of the EAS [35,37], but also mucin pool inclusions with histiocytes [34]. In rabbits, Rajasekaran investigated the effects of an EAS clear-cut section over time. The authors reported early collagen deposits from one week after injury, but also extensive fibrosis appearing three weeks after injury, at the site of myotomy but also beyond [61]. The presence of fibrosis was confirmed by other authors in rabbits [40,43]. In dogs, three weeks after IAS and EAS partial excision, Kang et al. reported focal interstitial inflammation, fibrosis and atrophy of smooth and striated muscles [46].

Anal sphincters encircle the anal canal, and the loss of this circular shape, as a result of injury, directly impairs the continence function [62]. However, during sphincter repair surgery, one can observe that the retracted muscle edges are held together with fibrous tissue, which bridges the defect [63]. Thus, unrepaired damage to IAS or EAS does not evolve into a hole in the sphincter ring (EAS and/or IAS), but rather into altered tissue, which fills the breach. In rats, this tissue contained mast cells and other inflammatory cells [18]. To the best of our knowledge, the importance of this tissue was never investigated in humans but could be of importance, especially if the muscle gap is the target of cell therapy.

Another strategy to treat patients with ASI is the transplantation of a biosphincter. Bitar’s group, from the Wake Forest Institute for Regenerative Medicine, has been working for decades on the implantation of bioengineered anal sphincters composed of innervated smooth muscle cells (Table S2). The results are promising and the group recently implanted the construct in large animals, including non-human primates [47,48]. This strategy is interesting as it provides a complete and functional IAS substitute and might be part of a reconstruction strategy for patients in whom IAS has been totally or partially removed such as for ultra-low rectal cancer resections. However, IAS engineering has some hurdles to overcome. First, this approach uses IAS or digestive smooth muscle cells and intestinal neuronal cells. For translation to the clinic, tissue might be procured from organ donors, requiring further immunosuppression and exposing patients to its associated risks. Those risks need to be balanced with the fact that ASI is a non-life-threatening condition. Moreover, Araki et al. recently demonstrated the feasibility of anorectal transplantation in a dog model, with this approach being serious concurrent to allogenic biosphincters [70]. Further, the tissue culture of such constructs requires 6 to 8 weeks of culture, which increases the risk of microbial contamination. Finally, the cost of the creation of an organic construct seems to be higher than cell isolation and expansion.

References from *Subsection 2.4. Secondary Repaired Anal Sphincter Injury* were updated from:

Patients with visible EAS or IAS sphincter lesions are good candidates for surgical repair. Park’s sphincteroplasty is the most common procedure where the two edges of the damaged sphincters are brought together with an overlapping suture [5,63]. Usually, the surrounding fibrous tissue, which also connects the retracted muscle, is not dissected as it offers a firm support for knotting [63]. To the best of our knowledge, the healing of such a delayed repair has never been investigated. Due to the poor long-term results of sphincteroplasty, the idea to strengthen the reparation with multipotent cells has emerged. As mentioned above, in preclinical studies, some authors injected multipotent cells during sphincter repair surgery but this was never done after secondary repair in animals [28,29,32,33,41]. However, Sarveazad et al. successfully and safely injected adipose tissue-derived multipotent cells after EAS sphincteroplasty in five women and two men (Table 2) [71].

To:

Patients with visible EAS or IAS sphincter lesions are good candidates for surgical repair. Park’s sphincteroplasty is the most common procedure where the two edges of the damaged sphincters are brought together with an overlapping suture [5,63]. Usually, the surrounding fibrous tissue, which also connects the retracted muscle, is not dissected as it offers a firm support for knotting [63]. To the best of our knowledge, the healing of such a delayed repair has never been investigated. Due to the poor long-term results of sphincteroplasty, the idea to strengthen the reparation with multipotent cells has emerged. As mentioned above, in preclinical studies, some authors injected multipotent cells during sphincter repair surgery but this was never done after secondary repair in animals [20,21,24,25,33]. However, Sarveazad et al. successfully and safely injected adipose tissue-derived multipotent cells after EAS sphincteroplasty in five women and two men (Table 2) [71].

References from Section 3, *Subsection 3.1. Multipotent Cell Origins*, Paragraphs 1–5, were updated from:

Multipotent cells proposed for ASI cell therapy can be derived from various tissues. The most frequent sites were skeletal muscle, bone marrow or adipose tissue. Cell preparations were either syngeneic or autologous, and allogeneic or xenogeneic transplantation was marginal (four preclinical studies and one clinical study). Cells from a muscular origin were used in the majority of the identified publications: In 17 studies, they originated from skeletal muscle or EAS [32,33,36,37,49,50,52–54,66–69,73,74,91–93] and in 11 studies, from smooth muscle or IAS [56,79,94–102] (Table 3). Four out of six clinical trials used skeletal muscle multipotent cells whereas the other used adipose tissue multipotent cells [66–69,74]. Two in vivo publications reported the use of commercial H9c2 rat heart myoblasts [28,42].

Cells of bone marrow origin were used in 10 studies [5,26,27,30,39–41,43,46,48] and only six tested cells originating from adipose tissue [31,34,35,38,71,72]. Neural cells were used for bioengineered constructs in eight publications [92–99,102,103]. Bioengineered constructs used smooth muscle seeded with neuronal cells from different origins. Finally, only one publication evaluated the potential of human umbilical cord matrix cells [48].

Skeletal muscle-derived cells seem to be an interesting source for ASI therapy. Different multipotent cells can be extracted from skeletal muscle: Satellite cells and other resident multipotent cells, the former having the ability to become new satellite cells or myoblasts, the precursors of myocytes (Figure 1) [104]. Thurner et al. demonstrated that smooth muscle cells can eventually be derived from myogenic progenitors [105]. The function of other muscle resident multipotent cells is not completely understood and this category encompasses different kinds of multipotent cells such as adult pericytes, PW1+ interstitial cells and fibro-adipogenic progenitors [106].

Satellite cells have the ability to reform muscle fiber [104] and have been proposed to treat conditions such as Duchenne disease [107], coronary artery disease [108] and also urinary incontinence [109]. It is supposed that skeletal muscle multipotent cells differentiated into new myocyte and could resupply EAS with new fibers (Figure 1). Indeed, the apparition of new fibers in EAS and the expression of muscle proteins have been observed by several authors [36,50,52,53,91,92]. However, the appearance of new muscle fibers does not necessarily indicate that anal function is improved. In this regard, several in vivo studies [28,37,54] using myogenic cells were inconclusive concerning anal function recovery despite cell engraftment confirmation.

Cells derived from adipose tissue constitute an interesting option, as subcutaneous fat tissue is easily accessible. The cells used in the reported studies [31,34,35,71] had the characteristics of MSC including the ability to differentiate into various tissues. MSC are multipotent cells that have been evaluated over the last years to treat various conditions including spinal cord injury, corneal or uvea injury, lung injury, cerebral injury, colitis, alopecia, muscular degenerative disease, myocardial infarction, liver injury, multiple sclerosis, Parkinson disease, cancer and to improve wound healing [110]. Indeed, allogeneic adipose tissue MSCs became popular because of their poor immunogenicity and their availability after liposuction surgery. However, recent evidence demonstrated the development of donor-specific antibodies, and MSC rejection has been documented. Besides MSC’s ability to differentiate into various cells, their paracrine action have been proposed to mediate most of their effects [110,111]. MSC produce a large amount of growth factors and extracellular vesicles [112]. Strategies using encapsulated MSC conserve the effects of MSC, notably on liver fibrosis, confirming the efficacy of paracrine action and treatment [113]. Thus, MSC can be seen as in situ bioreactors delivering growth factors to neighboring cells. MSC have the ability to induce smooth muscle regeneration from the gut and the bladder [114,115] and also skeletal muscle regeneration [116,117]. Thus, MSC therapy may promote the healing and regeneration of both IAS and EAS.

To:

Multipotent cells proposed for ASI cell therapy can be derived from various tissues. The most frequent sites were skeletal muscle, bone marrow or adipose tissue. Cell preparations were either syngeneic or autologous, and allogeneic or xenogeneic transplantation was marginal (four preclinical studies and one clinical study). Cells from a muscular origin were used in the majority of the identified publications: In 17 studies, they originated from skeletal muscle or EAS [24,25,28,29,41,42,44–46,66–69,74,91–93] and in 11 studies, from smooth muscle or IAS [48,79,94–102] (Table 3). Four out of six clinical trials used skeletal muscle multipotent cells whereas the other used adipose tissue multipotent cells [66–69,74]. Two in vivo publications reported the use of commercial H9c2 rat heart myoblasts [20,34].

Cells of bone marrow origin were used in nine studies [15,18,19,22,31,33,36,38,40] and only six tested cells originating from adipose tissue [23,26,27,30,71,72]. Neural cells were used for bioengineered constructs in eight publications [95–99,102]. Bioengineered constructs used smooth muscle seeded with neuronal cells from different origins. Finally, only one publication evaluated the potential of human umbilical cord matrix cells [40].

Skeletal muscle-derived cells seem to be an interesting source for ASI therapy. Different multipotent cells can be extracted from skeletal muscle: Satellite cells and other resident multipotent cells, the former having the ability to become new satellite cells or myoblasts, the precursors of myocytes (Figure 1) [104]. Thurner et al. demonstrated that smooth muscle cells can eventually be derived from myogenic progenitors [105]. The function of other muscle resident multipotent cells is not completely understood and this category encompasses different kinds of multipotent cells such as adult pericytes, PW1+ interstitial cells and fibro-adipogenic progenitors [106].

Satellite cells have the ability to reform muscle fiber [104] and have been proposed to treat conditions such as Duchenne disease [107], coronary artery disease [108] and also urinary incontinence [109]. It is supposed that skeletal muscle multipotent cells differentiated into new myocyte and could resupply EAS with new fibers (Figure 1). Indeed, the apparition of new fibers in EAS and the expression of muscle proteins have been observed by several authors [28,42,44,45,91,92]. However, the appearance of new muscle fibers does not necessarily indicate that anal function is improved. In this regard, several in vivo studies [20,29,46] using myogenic cells were inconclusive concerning anal function recovery despite cell engraftment confirmation.

Cells derived from adipose tissue constitute an interesting option, as subcutaneous fat tissue is easily accessible. The cells used in the reported studies [23,26,27,71] had the characteristics of MSC including the ability to differentiate into various tissues. MSC are multipotent cells that have been evaluated over the last years to treat various conditions including spinal cord injury, corneal or uvea injury, lung injury, cerebral injury, colitis, alopecia, muscular degenerative disease, myocardial infarction, liver injury, multiple sclerosis, Parkinson disease, cancer and to improve wound healing [110]. Indeed, allogeneic adipose tissue MSCs became popular because of their poor immunogenicity and their availability after liposuction surgery. However, recent evidence demonstrated the development of donor-specific antibodies, and MSC rejection has been documented [111]. Besides MSC’s ability to differentiate into various cells, their paracrine action have been proposed to mediate most of their effects [110,112]. MSC produce a large amount of growth factors and extracellular vesicles [113]. Strategies using encapsulated MSC conserve the effects of MSC, notably on liver fibrosis, confirming the efficacy of paracrine action and treatment [114]. Thus, MSC can be seen as in situ bioreactors delivering growth factors to neighboring cells. MSC have the ability to induce smooth muscle regeneration from the gut and the bladder [115,116] and also skeletal muscle regeneration [117,118]. Thus, MSC therapy may promote the healing and regeneration of both IAS and EAS.

References from *Subsection 3.2.* and *Subsection 3.3.*, were updated from:

*3.2. Methods for Multipotent Cell Isolation and Processing*

Stem cell and progenitor cells were retrieved from rats, mice, rabbits, non-human primate or humans (Table S1). Different harvest methods were used according to the origin of the collected tissue, but protocols were similar to existing standard, with the first step of washing and decontamination followed by the digestion of tissue and, finally, the purification of the cell suspension before plating. In the majority of studies, isolation procedures were sufficiently detailed, but some studies lack essential information concerning the isolation procedures. Skeletal muscle was digested with collagenase I [49,50,98], collagenase type II [93], collagenase type IV [92] collagenase type XI [37,54,91], collagenase NB6 [69], trypsin [91] and and/or dispase II [52,53,91]. Intestinal smooth muscle was digested using collagenase I [94] or collagenase II [79,93,95,97,99–103,119]. Enteric neurons were isolated after the digestion of tissue with collagenase II and dispase II [15,95,97,102,103]. For bone-marrow MSC, bones were flushed, and bone marrow collected, washed, sometimes fractionated with density gradient and plated [27,39,40,43–46]. For fat multipotent cells, adipose tissue was digested with collagenase I [31,34,35,71].

*3.3. Methods for Multipotent Cell Characterization*

The characterization of isolated multipotent cells is mandatory for reproducibility but also for quality purpose, especially when a clinical application for ASI treatment is foreseen. Among publications using muscle multipotent cells, only 13 studies reported or referred to a proper characterization of the cells and were heterogeneous for markers (Table 3) [36,37,49,50,52–54,56,66–69,93]. Indeed, international criteria for MSC definition were not always applied/fulfilled/verified [120].

Cells of bone marrow origin were used in nine publications [15,26,27,30,39,41,43,46,48] and were well characterized only in seven [15,26,27,39,41,43,46]. Cells originating from adipose tissue were more often precisely characterized and were at least CD90^+^ and CD45^−^ [31,34,35,38,71]. Satellite cells have typical features such as the expression of the transcription factor PAX7 (Figure 1) [104] but there is currently a lack of standardization in the nomenclature and characterization of other myogenic cells [121]. Thus, multipotent cells from a skeletal origin were widely used to treat in vivo models of ASI, but their efficacy, as well as their identification, remained elusive. As the cell types used were insufficiently characterized, it cannot be excluded that the beneficial effect on ASI was partially mediated by co-isolated contaminant multipotent cells from connective tissue. Moreover, if we assume that satellite cells were responsible for the positive effects of skeletal muscle cell preparation on ASI, this effect was thus induced by their action on the skeletal muscle of EAS and not on the smooth muscle of IAS.

To:

*3.2. Methods for Multipotent Cell Isolation and Processing*

Stem cell and progenitor cells were retrieved from rats, mice, rabbits, non-human primate or humans (Table S1). Different harvest methods were used according to the origin of the collected tissue, but protocols were similar to existing standard, with the first step of washing and decontamination followed by the digestion of tissue and, finally, the purification of the cell suspension before plating. In the majority of studies, isolation procedures were sufficiently detailed, but some studies lack essential information concerning the isolation procedures. Skeletal muscle was digested with collagenase I [41,42,98], collagenase type II [93], collagenase type IV [92] collagenase type XI [29,46,91], collagenase NB6 [69], trypsin [91] and and/or dispase II [44,45,91]. Intestinal smooth muscle was digested using collagenase I [94] or collagenase II [79,93,95,97,99–103,119]. Enteric neurons were isolated after the digestion of tissue with collagenase II and dispase II [15,95,97,102,103]. For bone-marrow MSC, bones were flushed, and bone marrow collected, washed, sometimes fractionated with density gradient and plated [18,31,32,35–38]. For fat multipotent cells, adipose tissue was digested with collagenase I [23,26,27,71].

*3.3. Methods for Multipotent Cell Characterization*

The characterization of isolated multipotent cells is mandatory for reproducibility but also for quality purpose, especially when a clinical application for ASI treatment is foreseen. Among publications using muscle multipotent cells, only 13 studies reported or referred to a proper characterization of the cells and were heterogeneous for markers (Table 3) [28,29,41,42,44–46,48,66–69,93]. Indeed, international criteria for MSC definition were not always applied/fulfilled/verified [120].

Cells of bone marrow origin were used in nine publications [15,18,19,22,31,33,36,40,118] and were well characterized only in seven [15,18,19,31,33,35,38]. Cells originating from adipose tissue were more often precisely characterized and were at least CD90^+^ and CD45^−^ [23,26,27,30,71]. Satellite cells have typical features such as the expression of the transcription factor PAX7 (Figure 1) [104] but there is currently a lack of standardization in the nomenclature and characterization of other myogenic cells [121]. Thus, multipotent cells from a skeletal origin were widely used to treat in vivo models of ASI, but their efficacy, as well as their identification, remained elusive. As the cell types used were insufficiently characterized, it cannot be excluded that the beneficial effect on ASI was partially mediated by co-isolated contaminant multipotent cells from connective tissue. Moreover, if we assume that satellite cells were responsible for the positive effects of skeletal muscle cell preparation on ASI, this effect was thus induced by their action on the skeletal muscle of EAS and not on the smooth muscle of IAS.

References from Section 4, *Subsections 4.1–4.4.* were updated from:

*4.1. Practical Considerations*

Before transplantation, cells were cultivated on plastic dishes and the number of passages before injection in the identified clinical trials ranged from three to ten. The number of injected cells, clearly reported by identified in vivo reports, ranged from 10,000 up to 90 million. In clinical trials, this number ranged from 200,000 up to 2 billion. However, the minimum number of cells required to obtain a beneficial effect on ASI remains elusive and only a few authors performed a real titration [36,92]. Thus, an excess of cells was used to compensate stem cell and progenitor cell death. Transplanted cell survival is a main concern in the field of cell therapy and it can be impaired by several factors related to the mechanical force applied during cell application, detachment from cell substrate and receiving site with inflammation and/or local hypoxia [122]. Indeed, forces generated during injection with a syringe needle are sufficient to induce up to 40% of cell death [122]. Thus, strategies have been developed to improve cell engraftment and transplantation success.

*4.2. Adjuvant Therapy*

As a strategy to limit cellular stress due to transplantation, some authors proposed to protect cells with biomaterials [52–54,110]. Indeed, it is known that preserving cell-extracellular substrate interactions can limit stem cell apoptosis [122]. Biomaterials were typically scaffolds of decellularized matrices or hydrogel polymers [31,41–43,51]. Deserving the same purpose, multipotent cells were also transplanted as sheets of cells instead of individual cells [34]. Alternatively, Trébol et al. seeded suture thread with MSC to be used for sphincter reconstruction [35] whereas Ding et al. reinforced reconstruction with a patch of an acellular dermal matrix also seeded with MSC [41].

As mentioned earlier, in some studies, authors performed immediate injection of cells along with sphincter repair, confronting cells with an acute inflammatory environment that presumably precludes cell survival [122]. On the other hand, inflammation can also enhance cell settling and homing, and the injection of myoblasts in healthy regions of EAS did not restore continence compared to the injection of cells into injured parts [36,122]. Other known strategies to improve cell survival implicated pre-conditioning of cells with either thermal preconditioning, hypoxic preconditioning, acidic preconditioning or nutrient deprivation preconditioning [122]. The goal of these strategies is to induce anti-apoptotic protein expression [122]. Injection sites were also prepared, and electrical stimulation was used in two in vivo studies [45,46] and two clinical studies to promote the homing of cells into anal sphincters [66,68]. Moreover, laser beam stimulation along with cell therapy was used in one study to promote muscle proliferation [38].

Further, growth factors (SDF-1, FGF) described to promote stem cell implantation were delivered in situ by bioscaffolds or through osmotic pumps [44,45,99,100,103]. Two studies transfected cells with SDF-1 plasmids before implantation [43,44]. As an alternative to the use of growth factors, platelet rich plasma, which is known to contain numerous growth factors, might be transplanted conjointly with stem cells [123].

*4.3. Measure of Outcomes and Results*

For in vitro studies, physiological functional evaluation was carried out in almost all identified studies to assess the contractility potential of constructed sphincters. For in vivo studies, outcomes were highly variable: The most common methods for outcome assessment were histology, anorectal manometry, physiological functionality evaluation and electromyography or electrophysiology. Some authors tracked cells using magnetic resonance imaging [50] or labeled them with fluorescent proteins (GFP) [91].

The determination of the outcome in a clinical study on anal incontinence is challenging. Different definitions of ASI exists, including incontinence to gases or not [1]. Further, in order to be comparable to the literature, authors are choosing outcomes that appear to be a gold standard in the medical literature. In almost all published clinical studies, one incontinence score was used as the primary outcome. However, a recent analysis of different incontinence scores pointed out that no single score reaches relevant psychometric soundness and recommended the use of at least two scores to evaluate ASI [123]. However, the utilization of objective outcomes may be more reliable such as high-resolution anal manometry or contact EMG (For e.g., MAPLe^®^ device, Medtronic, Dublin, Ireland [124]).

*4.4. Results*

A review of the literature identified a total of 52 original publications. Seven publications reported in vitro results (Table S2) [79,93–95,97,98], with six on bioengineered constructs among them [79,94,95,97,98]. One publication reported the isolation of cells from human IAS and EAS and assessed their viability [93]. In vivo experiments were reported in 38 publications (Table S1) [15,26,27,29–43,45,46,48–54,56,91,92,96,99–103], including five articles on heterotopic sphincter bioconstruct implantation [96,99–101,103]. Seven human studies were identified (Table 2) [66,68,69,71,72,74], including three randomized controlled trials [69,71,72] and one case report [74]. A total of 83 patients received cell therapy for ASI treatment.

Almost all patients included in clinical trials exhibited EAS injury. Four out of seven studies used the Wexner score as the primary outcome. The FIQL score was used in two studies. Other variables measured were anorectal manometry (5/7), endoanal ultrasonography (3/7) and electromyography (EMG) (3/7). The longest follow-up was reported by the group of Frudinger et al. who injected autologous myoblasts into EAS in 10 voluntary women with EAS defect or atrophy [66]. After a follow-up of 5 years, the mean Wexner score decreased from 15.3 (SD-2.4) before intervention to 0.7 (SD 1.3) (*p* > 0.001). In addition, anal manometry demonstrated an improvement of median resting and squeeze pressures (20 (IQR 17–28) to 32 (25–43) and 23 (IQR 20–34) to 33 (IQR 31–66), respectively). The same group started a second trial including 34 females and 5 males and found a reduction of Wexner score of −16.2 (SD-3.66) for women and −18.8 (SD-1.30) for male at one year. These results were better than sphincteroplasty, which typically induces a long-term reduction of −1 to −5.2 of median/mean Wexner score (mean follow-up between seven and eight years) [125,126]. In a similar study (muscle tissue-derived multipotent cells injected into EAS), Boyer et al. reported a reduction of median Wexner score of −6.4 (range −12 to 2) (*p* = 0.006) for the intervention group and a reduction of −1 (range −8 to 6) (*p* = 0.35) for the placebo [69]. However, using adipose tissue-derived multipotent cells in a randomized triple blinded placebo-controlled trial, De la Portilla et al. failed to demonstrate any effect on Wexner score of cell transplantation into EAS defect [72]. Globally, all seven reports described encouraging results regarding at least one of the measured outcomes (which was not necessary the primary outcome).

Until now, in vivo experiments have demonstrated a relative oncological safety of such a strategy. One in vivo study described focal cell growth at the injection site but without malignant characteristics [33]. Recent evidence confirmed that pure stem cell cultures, particularly MSC, did not develop malignant cells [127,128]. However, MSC have opposite effects on tumor cells and can promote or suppress tumor growth in vitro and in vivo [129]. Published clinical trials, which used either skeletal muscle tissue or adipose tissue multipotent cells, demonstrated that the procedures were safe.

To:

*4.1. Practical Considerations*

Before transplantation, cells were cultivated on plastic dishes and the number of passages before injection in the identified clinical trials ranged from three to ten. The number of injected cells, clearly reported by identified in vivo reports, ranged from 10,000 up to 90 million. In clinical trials, this number ranged from 200,000 up to 2 billion. However, the minimum number of cells required to obtain a beneficial effect on ASI remains elusive and only a few authors performed a real titration [28,92]. Thus, an excess of cells was used to compensate stem cell and progenitor cell death. Transplanted cell survival is a main concern in the field of cell therapy and it can be impaired by several factors related to the mechanical force applied during cell application, detachment from cell substrate and receiving site with inflammation and/or local hypoxia [122]. Indeed, forces generated during injection with a syringe needle are sufficient to induce up to 40% of cell death [122]. Thus, strategies have been developed to improve cell engraftment and transplantation success.

*4.2. Adjuvant Therapy*

As a strategy to limit cellular stress due to transplantation, some authors proposed to protect cells with biomaterials [44–46,110]. Indeed, it is known that preserving cell-extracellular substrate interactions can limit stem cell apoptosis [122]. Biomaterials were typically scaffolds of decellularized matrices or hydrogel polymers [23,33–35,43]. Deserving the same purpose, multipotent cells were also transplanted as sheets of cells instead of individual cells [26]. Alternatively, Trébol et al. seeded suture thread with MSC to be used for sphincter reconstruction [27] whereas Ding et al. reinforced reconstruction with a patch of an acellular dermal matrix also seeded with MSC [33].

As mentioned earlier, in some studies, authors performed immediate injection of cells along with sphincter repair, confronting cells with an acute inflammatory environment that presumably precludes cell survival [122]. On the other hand, inflammation can also enhance cell settling and homing, and the injection of myoblasts in healthy regions of EAS did not restore continence compared to the injection of cells into injured parts [28,122]. Other known strategies to improve cell survival implicated pre-conditioning of cells with either thermal preconditioning, hypoxic preconditioning, acidic preconditioning or nutrient deprivation preconditioning [122]. The goal of these strategies is to induce anti-apoptotic protein expression [122]. Injection sites were also prepared, and electrical stimulation was used in two in vivo studies [37,38] and two clinical studies to promote the homing of cells into anal sphincters [66,68]. Moreover, laser beam stimulation along with cell therapy was used in one study to promote muscle proliferation [30].

Further, growth factors (SDF-1, FGF) described to promote stem cell implantation were delivered in situ by bioscaffolds or through osmotic pumps [35,37,99,100,103]. Two studies transfected cells with SDF-1 plasmids before implantation [35,36]. As an alternative to the use of growth factors, platelet rich plasma, which is known to contain numerous growth factors, might be transplanted conjointly with stem cells [123].

*4.3. Measure of Outcomes and Results*

For in vitro studies, physiological functional evaluation was carried out in almost all identified studies to assess the contractility potential of constructed sphincters. For in vivo studies, outcomes were highly variable: The most common methods for outcome assessment were histology, anorectal manometry, physiological functionality evaluation and electromyography or electrophysiology. Some authors tracked cells using magnetic resonance imaging [42] or labeled them with fluorescent proteins (GFP) [91].

The determination of the outcome in a clinical study on anal incontinence is challenging. Different definitions of ASI exists, including incontinence to gases or not [1]. Further, in order to be comparable to the literature, authors are choosing outcomes that appear to be a gold standard in the medical literature. In almost all published clinical studies, one incontinence score was used as the primary outcome. However, a recent analysis of different incontinence scores pointed out that no single score reaches relevant psychometric soundness and recommended the use of at least two scores to evaluate ASI [124]. However, the utilization of objective outcomes may be more reliable such as high-resolution anal manometry or contact EMG (For e.g., MAPLe^®^ device, Medtronic, Dublin, Ireland [125]).

*4.4. Results*

A review of the literature identified a total of 52 original publications. Seven publications reported in vitro results (Table S2) [79,93–95,97,98,119], with six on bioengineered constructs among them [79,93–95,97,98]. One publication reported the isolation of cells from human IAS and EAS and assessed their viability [93]. In vivo experiments were reported in 38 publications (Table S1) [15,18,19,21–35,37,38,40–46,48,91,92,96,99–103], including five articles on heterotopic sphincter bioconstruct implantation [96,99–101,103]. Seven human studies were identified (Table 2) [66–69,71,72,74], including three randomized controlled trials [69–72] and one case report [74]. A total of 83 patients received cell therapy for ASI treatment.

Almost all patients included in clinical trials exhibited EAS injury. Four out of seven studies used the Wexner score as the primary outcome. The FIQL score was used in two studies. Other variables measured were anorectal manometry (5/7), endoanal ultrasonography (3/7) and electromyography (EMG) (3/7). The longest follow-up was reported by the group of Frudinger et al. who injected autologous myoblasts into EAS in 10 voluntary women with EAS defect or atrophy [66]. After a follow-up of 5 years, the mean Wexner score decreased from 15.3 (SD 2.4) before intervention to 0.7 (SD 1.3) (*p* > 0.001). In addition, anal manometry demonstrated an improvement of median resting and squeeze pressures (20 (IQR 17–28) to 32 (25–43) and 23 (IQR 20–34) to 33 (IQR 31–66), respectively). The same group started a second trial including 34 females and 5 males and found a reduction of Wexner score of −16.2 (SD-3.66) for women and −18.8 (SD-1.30) for male at one year. These results were better than sphincteroplasty, which typically induces a long-term reduction of −1 to −5.2 of median/mean Wexner score (mean follow-up between seven and eight years) [126,127]. In a similar study (muscle tissue-derived multipotent cells injected into EAS), Boyer et al. reported a reduction of median Wexner score of −6.4 (range −12 to 2) (*p* = 0.006) for the intervention group and a reduction of −1 (range −8 to 6) (*p* = 0.35) for the placebo [69]. However, using adipose tissue-derived multipotent cells in a randomized triple blinded placebo-controlled trial, De la Portilla et al. failed to demonstrate any effect on Wexner score of cell transplantation into EAS defect [72]. Globally, all seven reports described encouraging results regarding at least one of the measured outcomes (which was not necessary the primary outcome).

Until now, in vivo experiments have demonstrated a relative oncological safety of such a strategy. One in vivo study described focal cell growth at the injection site but without malignant characteristics [25]. Recent evidence confirmed that pure stem cell cultures, particularly MSC, did not develop malignant cells [128,129]. However, MSC have opposite effects on tumor cells and can promote or suppress tumor growth in vitro and in vivo [130]. Published clinical trials, which used either skeletal muscle tissue or adipose tissue multipotent cells, demonstrated that the procedures were safe.

References from the Conclusions, in Paragraphs 2 and 3, were updated from:

The ideal therapy for ASI should be cost-effective with a long-lasting effect. Apart from research and development costs, good manufacturing practices, GMP certifications and implementation charges, routine use of cell therapy appears to be highly costly [130]. Trébol et al. estimated the maximal production costs in Spain to be 7400 USD for 40 million autologous fat-derived cells or 8500 € for 100 million allogeneic fat-derived cells [129]. In our hospital, the cost for a sphincteroplasty, with a typical length of stay of two days, is 5000 USD. Recently, Gräs et al. proposed a cost-effective alternative to cell transplantation for anal sphincter regeneration. Following promising results for urinary incontinence, the authors discussed the possibility to inject fragmented muscle fibers, instead of expanded cells, into injured anal sphincters [121].

After transplantation, progenitor cells and stem cells might act by paracrine effects and/or by differentiation into functional muscular cells. It should be pointed out that the exact underlying mechanism remains poorly understood, and that basic research on this topic is still required to understand which factors and conditions are leading to cell engraftment, differentiation and finally tissue regeneration [131,132]. Moreover, the natural history of sphincter lesion/repaired sphincter healing should be better understood in order to select appropriate cell preparations and transplantation techniques. Some groups reported a different approach, considering the use of cell therapy as an add-on to sphincter repair, either directly after lesion, to simulate the primary repair of an acute obstetrical tear or at distance [26–33]. Thus, it remains to be determined how stem cells and progenitor cells should be used for ASI: As a substitution to surgery or along with surgery.

To:

The ideal therapy for ASI should be cost-effective with a long-lasting effect. Apart from research and development costs, good manufacturing practices, GMP certifications and implementation charges, routine use of cell therapy appears to be highly costly [131]. Trébol et al. estimated the maximal production costs in Spain to be 7400 USD for 40 million autologous fat-derived cells or 8500 € for 100 million allogeneic fat-derived cells [130]. In our hospital, the cost for a sphincteroplasty, with a typical length of stay of two days, is 5000 USD. Recently, Gräs et al. proposed a cost-effective alternative to cell transplantation for anal sphincter regeneration. Following promising results for urinary incontinence, the authors discussed the possibility to inject fragmented muscle fibers, instead of expanded cells, into injured anal sphincters [121].

After transplantation, progenitor cells and stem cells might act by paracrine effects and/or by differentiation into functional muscular cells. It should be pointed out that the exact underlying mechanism remains poorly understood, and that basic research on this topic is still required to understand which factors and conditions are leading to cell engraftment, differentiation and finally tissue regeneration [123,132]. Moreover, the natural history of sphincter lesion/repaired sphincter healing should be better understood in order to select appropriate cell preparations and transplantation techniques. Some groups reported a different approach, considering the use of cell therapy as an add-on to sphincter repair, either directly after lesion, to simulate the primary repair of an acute obstetrical tear or at distance [18–25]. Thus, it remains to be determined how stem cells and progenitor cells should be used for ASI: As a substitution to surgery or along with surgery.

The references from the first phrase of Figure 1 caption were corrected from:

Schematic representation of events occurring after delivery-related acute anal sphincters injury [20,22].

To:

Schematic representation of events occurring after delivery-related acute anal sphincters injury [51,53].

The list of updated and rearranged references is the following:

1. Chatoor, D.R.; Taylor, S.J.; Cohen, C.R.G.; Emmanuel, A.V. Faecal Incontinence. *Br. J. Surg.* **2007**, *94*, 134–144. https://doi.org/10.1002/bjs.5676.
2. Meyer, I.; Richter, H.E. Impact of Fecal Incontinence and Its Treatment on Quality of Life in Women. *Womens Health Lond. Engl.* **2015**, *11*, 225–238. https://doi.org/10.2217/whe.14.66.
3. Williams, K.S.; Shalom, D.F.; Winkler, H.A. Faecal Incontinence: A Narrative Review of Clinic-Based Management for the General Gynaecologist. *J. Obstet. Gynaecol. J. Inst. Obstet. Gynaecol.* **2018**, *38*, 1–9. https://doi.org/10.1080/01443615.2017.1344204.
4. Nandivada, P.; Nagle, D. Surgical Therapies for Fecal Incontinence. *Curr. Opin. Gastroenterol.* **2014**, *30*, 69–74. https://doi.org/10.1097/MOG.0000000000000029.
5. Wexner, S.D.; Bleier, J. Current Surgical Strategies to Treat Fecal Incontinence. *Expert Rev. Gastroenterol. Hepatol.* **2015**, *9*, 1577–1589. https://doi.org/10.1586/17474124.2015.1093417.
6. Zorcolo, L.; Covotta, L.; Bartolo, D.C.C. Outcome of Anterior Sphincter Repair for Obstetric Injury: Comparison of Early and Late Results. *Dis. Colon Rectum* **2005**, *48*, 524–531. https://doi.org/10.1007/s10350-004-0770-1.
7. Gronewold, M.; Kroencke, T.; Hagedorn, A.; Tunn, R.; Gauruder-Burmester, A. [External anal sphincter repair using the overlapping technique in patients with anal incontinence and concomitant pudendal nerve damage]. *Zentralblatt Für Chir.* **2008**, *133*, 129–134. https://doi.org/10.1055/s-2008-1004734.
8. Demirbas, S.; Atay, V.; Sucullu, I.; Filiz, A.I. Overlapping Repair in Patients with Anal Sphincter Injury. *Med. Princ. Pract. Int. J. Kuwait Univ. Health Sci. Cent.* **2008**, *17*, 56–60. https://doi.org/10.1159/000109591.
9. Brown, S.R.; Wadhawan, H.; Nelson, R.L. Surgery for Faecal Incontinence in Adults. *Cochrane Database Syst. Rev.* **2013**, CD001757. https://doi.org/10.1002/14651858.CD001757.pub4.
10. Zutshi, M.; Hull, T.; Gurland, B. Anal Encirclement with Sphincter Repair (AESR Procedure) Using a Biological Graft for Anal Sphincter Damage Involving the Entire Circumference. *Colorectal Dis. Off. J. Assoc. Coloproctology G. B. Irel.* **2012**, *14*, 592–595. https://doi.org/10.1111/j.1463-1318.2011.02675.x.
11. De la Portilla, F. Internal Anal Sphincter Augmentation and Substitution. *Gastroenterol. Rep.* **2014**, *2*, 106–111. https://doi.org/10.1093/gastro/gou004.
12. Alam, N.N.; Narang, S.K.; Köckerling, F.; Daniels, I.R.; Smart, N.J. Anal Sphincter Augmentation Using Biological Material. *Front. Surg.* **2015**, *2*. https://doi.org/10.3389/fsurg.2015.00060.
13. Rao, S.S.C. Pathophysiology of Adult Fecal Incontinence. *Gastroenterology* **2004**, *126*, S14–S22.
14. LaCross, A.; Groff, M.; Smaldone, A. Obstetric Anal Sphincter Injury and Anal Incontinence Following Vaginal Birth: A Systematic Review and Meta-Analysis. *J. Midwifery Womens Health* **2015**, *60*, 37–47. https://doi.org/10.1111/jmwh.12283.
15. Salcedo, L.; Mayorga, M.; Damaser, M.; Balog, B.; Butler, R.; Penn, M.; Zutshi, M. Mesenchymal Stem Cells Can Improve Anal Pressures after Anal Sphincter Injury. *Stem Cell Res.* **2013**, *10*, 95–102. https://doi.org/10.1016/j.scr.2012.10.002.
16. Healy, C.F.; O’Herlihy, C.; O’Brien, C.; O’Connell, P.R.; Jones, J.F.X. Experimental Models of Neuropathic Fecal Incontinence: An Animal Model of Childbirth Injury to the Pudendal Nerve and External Anal Sphincter. *Dis. Colon Rectum* **2008**, *51*, 1619–1626; discussion 1626. https://doi.org/10.1007/s10350-008-9283-7.
17. Wai, C.Y.; Rahn, D.D.; White, A.B.; Word, R.A. Recovery of External Anal Sphincter Contractile Function after Prolonged Vaginal Distention or Sphincter Transection in an Animal Model. *Obstet. Gynecol.* **2008**, *111*, 1426–1434. https://doi.org/10.1097/AOG.0b013e318173f0b8.
18. Mazzanti, B.; Lorenzi, B.; Borghini, A.; Boieri, M.; Ballerini, L.; Saccardi, R.; Weber, E.; Pessina, F. Local Injection of Bone Marrow Progenitor Cells for the Treatment of Anal Sphincter Injury: In-Vitro Expanded versus Minimally-Manipulated Cells. *Stem Cell Res. Ther.* **2016**, *7*, 85. https://doi.org/10.1186/s13287-016-0344-x.
19. Lorenzi, B.; Pessina, F.; Lorenzoni, P.; Urbani, S.; Vernillo, R.; Sgaragli, G.; Gerli, R.; Mazzanti, B.; Bosi, A.; Saccardi, R.; et al. Treatment of Experimental Injury of Anal Sphincters with Primary Surgical Repair and Injection of Bone Marrow-Derived Mesenchymal Stem Cells. *Dis. Colon Rectum* **2008**, *51*, 411–420. https://doi.org/10.1007/s10350-007-9153-8.
20. Fitzwater, J.L.; Grande, K.B.; Sailors, J.L.; Acevedo, J.F.; Word, R.A.; Wai, C.Y. Effect of Myogenic Stem Cells on the Integrity and Histomorphology of Repaired Transected External Anal Sphincter. *Int. Urogynecol. J.* **2015**, *26*, 251–256. https://doi.org/10.1007/s00192-014-2496-5.
21. White, A.B.; Keller, P.W.; Acevedo, J.F.; Word, R.A.; Wai, C.Y. Effect of Myogenic Stem Cells on Contractile Properties of the Repaired and Unrepaired Transected External Anal Sphincter in an Animal Model. *Obstet. Gynecol.* **2010**, *115*, 815–823. https://doi.org/10.1097/AOG.0b013e3181d56cc5.
22. Pathi, S.D.; Acevedo, J.F.; Keller, P.W.; Kishore, A.H.; Miller, R.T.; Wai, C.Y.; Word, R.A. Recovery of the Injured External Anal Sphincter after Injection of Local or Intravenous Mesenchymal Stem Cells. *Obstet. Gynecol.* **2012**, *119*, 134–144. https://doi.org/10.1097/AOG.0b013e3182397009.
23. Kuismanen, K.; Juntunen, M.; Narra Girish, N.; Tuominen, H.; Huhtala, H.; Nieminen, K.; Hyttinen, J.; Miettinen, S. Functional Outcome of Human Adipose Stem Cell Injections in Rat Anal Sphincter Acute Injury Model. *Stem Cells Transl. Med.* **2018**, *7*, 295–304. https://doi.org/10.1002/sctm.17-0208.
24. Lane, F.L.; Jacobs, S.A.; Craig, J.B.; Nistor, G.; Markle, D.; Noblett, K.L.; Osann, K.; Keirstead, H. In Vivo Recovery of the Injured Anal Sphincter after Repair and Injection of Myogenic Stem Cells: An Experimental Model. *Dis. Colon Rectum* **2013**, *56*, 1290–1297. https://doi.org/10.1097/DCR.0b013e3182a4adfb.
25. Jacobs, S.A.; Lane, F.L.; Pham, Q.-A.; Nistor, G.; Robles, R.; Chua, C.; Boubion, B.; Osann, K.; Keirstead, H. Safety Assessment of Myogenic Stem Cell Transplantation and Resulting Tumor Formation. *Female Pelvic Med. Reconstr. Surg.* **2013**, *19*, 362–368. https://doi.org/10.1097/SPV.0000000000000035.
26. Inoue, Y.; Fujita, F.; Yamaguchi, I.; Kinoe, H.; Kawahara, D.; Sakai, Y.; Kuroki, T.; Eguchi, S. Improvement of Anal Function by Adipose-Derived Stem Cell Sheets. *Dig. Surg.* **2018**, *35*, 64–69. https://doi.org/10.1159/000475475.
27. Trébol, J.; Georgiev-Hristov, T.; Vega-Clemente, L.; García-Gómez, I.; Carabias-Orgaz, A.; García-Arranz, M.; García-Olmo, D. Rat Model of Anal Sphincter Injury and Two Approaches for Stem Cell Administration. *World J. Stem Cells* **2018**, *10*, 1–14. https://doi.org/10.4252/wjsc.v10.i1.1.
28. Bisson, A.; Fréret, M.; Drouot, L.; Jean, L.; Le Corre, S.; Gourcerol, G.; Doucet, C.; Michot, F.; Boyer, O.; Lamacz, M. Restoration of Anal Sphincter Function after Myoblast Cell Therapy in Incontinent Rats. *Cell Transplant.* **2015**, *24*, 277–286. https://doi.org/10.3727/096368913X674053.
29. Kang, S.-B.; Lee, H.N.; Lee, J.Y.; Park, J.-S.; Lee, H.S.; Lee, J.Y. Sphincter Contractility after Muscle-Derived Stem Cells Autograft into the Cryoinjured Anal Sphincters of Rats. *Dis. Colon Rectum* **2008**, *51*, 1367–1373. https://doi.org/10.1007/s10350-008-9360-y.
30. Sarveazad, A.; Babahajian, A.; Yari, A.; Rayner, C.K.; Mokhtare, M.; Babaei-Ghazani, A.; Agah, S.; Mahjoubi, B.; Shamseddin, J.; Yousefifard, M. Combination of Laser and Human Adipose-Derived Stem Cells in Repair of Rabbit Anal Sphincter Injury: A New Therapeutic Approach. *Stem Cell Res. Ther.* **2019**, *10*, 367. https://doi.org/10.1186/s13287-019-1477-5.
31. Salcedo, L.; Penn, M.; Damaser, M.; Balog, B.; Zutshi, M. Functional Outcome after Anal Sphincter Injury and Treatment with Mesenchymal Stem Cells. *Stem Cells Transl. Med.* **2014**, *3*, 760–767. https://doi.org/10.5966/sctm.2013-0157.
32. Li, P.; Ma, X.; Jin, W.; Li, X.; Hu, J.; Jiang, X.; Guo, X. Effects of Local Injection and Intravenous Injection of Allogeneic Bone Marrow Mesenchymal Stem Cells on the Structure and Function of Damaged Anal Sphincter in Rats. *J. Tissue Eng. Regen. Med.* **2020**, *14*, 989–1000. https://doi.org/10.1002/term.3079.
33. Ding, Z.; Liu, X.; Ren, X.; Zhang, Q.; Zhang, T.; Qian, Q.; Liu, W.; Jiang, C. Galectin-1-Induced Skeletal Muscle Cell Differentiation of Mesenchymal Stem Cells Seeded on an Acellular Dermal Matrix Improves Injured Anal Sphincter. *Discov. Med.* **2016**, *21*, 331–340.
34. Montoya, T.I.; Acevedo, J.F.; Smith, B.; Keller, P.W.; Sailors, J.L.; Tang, L.; Word, R.A.; Wai, C.Y. Myogenic Stem Cell-Laden Hydrogel Scaffold in Wound Healing of the Disrupted External Anal Sphincter. *Int. Urogynecol. J.* **2015**, *26*, 893–904. https://doi.org/10.1007/s00192-014-2620-6.
35. Sun, L.; Xie, Z.; Kuang, M.; Penn, M.; Damaser, M.S.; Zutshi, M. Regenerating the Anal Sphincter: Cytokines, Stem Cells, or Both? *Dis. Colon Rectum* **2017**, *60*, 416–425. https://doi.org/10.1097/DCR.0000000000000783.
36. Sun, L.; Kuang, M.; Penn, M.; Damaser, M.S.; Zutshi, M. Stromal Cell-Derived Factor 1 Plasmid Regenerates Both Smooth and Skeletal Muscle After Anal Sphincter Injury in the Long Term. *Dis. Colon Rectum* **2017**, *60*, 1320–1328. https://doi.org/10.1097/DCR.0000000000000940.
37. Sun, L.; Yeh, J.; Xie, Z.; Kuang, M.; Damaser, M.S.; Zutshi, M. Electrical Stimulation Followed by Mesenchymal Stem Cells Improves Anal Sphincter Anatomy and Function in a Rat Model at a Time Remote From Injury. *Dis. Colon Rectum* **2016**, *59*, 434–442. https://doi.org/10.1097/DCR.0000000000000548.
38. Li, X.; Guo, X.; Jin, W.; Lu, J. Effects of Electroacupuncture Combined with Stem Cell Transplantation on Anal Sphincter Injury-Induced Faecal Incontinence in a Rat Model. *Acupunct. Med. J. Br. Med. Acupunct. Soc.* **2018**, *36*, 254–260. https://doi.org/10.1136/acupmed-2016-011262.
39. Yamaguchi, I.; Fujita, F.; Yamanouchi, K.; Mishima, T.; Kawahara, D.; Sakai, Y.; Ito, S.; Kanetaka, K.; Takatsuki, M.; Kuroki, T.; et al. A Novel Animal Model of Long-Term Sustainable Anal Sphincter Dysfunction. *J. Surg. Res.* **2013**, *184*, 813–818. https://doi.org/10.1016/j.jss.2013.04.010.
40. Aghaee-Afshar, M.; Rezazadehkermani, M.; Asadi, A.; Malekpour-Afshar, R.; Shahesmaeili, A.; Nematollahi-mahani, S.N. Potential of Human Umbilical Cord Matrix and Rabbit Bone Marrow-Derived Mesenchymal Stem Cells in Repair of Surgically Incised Rabbit External Anal Sphincter. *Dis. Colon Rectum* **2009**, *52*, 1753–1761. https://doi.org/10.1007/DCR.0b013e3181b55112.
41. Kajbafzadeh, A.-M.; Kajbafzadeh, M.; Sabetkish, S.; Sabetkish, N.; Tavangar, S.M. Tissue-Engineered External Anal Sphincter Using Autologous Myogenic Satellite Cells and Extracellular Matrix: Functional and Histological Studies. *Ann. Biomed. Eng.* **2016**, *44*, 1773–1784. https://doi.org/10.1007/s10439-015-1468-3.
42. Elmi, A.; Kajbafzadeh, A.-M.; Oghabian, M.A.; Talab, S.S.; Tourchi, A.; Khoei, S.; Rafie, B.; Esfahani, S.A. Anal Sphincter Repair with Muscle Progenitor Cell Transplantation: Serial Assessment with Iron Oxide-Enhanced MRI. *AJR Am. J. Roentgenol.* **2014**, *202*, 619–625. https://doi.org/10.2214/AJR.13.11146.
43. Kajbafzadeh, A.-M.; Elmi, A.; Talab, S.S.; Esfahani, S.A.; Tourchi, A. Functional External Anal Sphincter Reconstruction for Treatment of Anal Incontinence Using Muscle Progenitor Cell Auto Grafting. *Dis. Colon Rectum* **2010**, *53*, 1415–1421. https://doi.org/10.1007/DCR.0b013e3181e53088.
44. Oh, H.-K.; Lee, H.S.; Lee, J.H.; Oh, S.H.; Lim, J.-Y.; Ahn, S.; Hwang, J.-Y.; Kang, S.-B. Functional and Histological Evidence for the Targeted Therapy Using Biocompatible Polycaprolactone Beads and Autologous Myoblasts in a Dog Model of Fecal Incontinence. *Dis. Colon Rectum* **2015**, *58*, 517–525. https://doi.org/10.1097/DCR.0000000000000346.
45. Oh, H.-K.; Lee, H.S.; Lee, J.H.; Oh, S.H.; Lim, J.-Y.; Ahn, S.; Kang, S.-B. Coadministration of Basic Fibroblast Growth Factor-Loaded Polycaprolactone Beads and Autologous Myoblasts in a Dog Model of Fecal Incontinence. *Int. J. Colorectal Dis.* **2015**, *30*, 549–557. https://doi.org/10.1007/s00384-015-2121-1.
46. Kang, S.-B.; Lee, H.S.; Lim, J.-Y.; Oh, S.H.; Kim, S.J.; Hong, S.-M.; Jang, J.-H.; Cho, J.-E.; Lee, S.-M.; Lee, J.H. Injection of Porous Polycaprolactone Beads Containing Autologous Myoblasts in a Dog Model of Fecal Incontinence. *J. Korean Surg. Soc.* **2013**, *84*, 216–224. https://doi.org/10.4174/jkss.2013.84.4.216.
47. Bitar, K.N.; Zakhem, E.; Bohl, J.L.; Tamburini, R.; Dadhich, P.; Scott, C.; Knutson, D.T.; Giliam, J.H. Implantation of Autologous Biosphincters in a Non-Human Primate (NHP) Model of Fecal Incontinence. *Gastroenterology* **2017**, *152*, S15.
48. Dadhich, P.; Bohl, J.L.; Tamburrini, R.; Zakhem, E.; Scott, C.; Kock, N.; Mitchell, E.; Gilliam, J.; Bitar, K.N. BioSphincters to Treat Fecal Incontinence in Nonhuman Primates. *Sci. Rep.* **2019**, *9*, 18096. https://doi.org/10.1038/s41598-019-54440-3.
49. Meister, M.R.; Rosenbloom, J.I.; Lowder, J.L.; Cahill, A.G. Techniques for Repair of Obstetric Anal Sphincter Injuries. *Obstet. Gynecol. Surv.* **2018**, *73*, 33–39. https://doi.org/10.1097/OGX.0000000000000521.
50. Mitoyan, L.; Chevrier, V.; Hernandez-Vargas, H.; Ollivier, A.; Homayed, Z.; Pannequin, J.; Poizat, F.; De Biasi-Cador, C.; Charafe-Jauffret, E.; Ginestier, C.; et al. A Stem Cell Population at the Anorectal Junction Maintains Homeostasis and Participates in Tissue Regeneration. *Nat. Commun.* **2021**, *12*, 2761. https://doi.org/10.1038/s41467-021-23034-x.
51. Plair, A.; Bennington, J.; Williams, J.K.; Parker-Autry, C.; Matthews, C.A.; Badlani, G. Regenerative Medicine for Anal Incontinence: A Review of Regenerative Therapies beyond Cells. *Int. Urogynecol. J.* **2020**. https://doi.org/10.1007/s00192-020-04620-x.
52. Sass, F.A.; Fuchs, M.; Pumberger, M.; Geissler, S.; Duda, G.N.; Perka, C.; Schmidt-Bleek, K. Immunology Guides Skeletal Muscle Regeneration. *Int. J. Mol. Sci.* **2018**, *19*, 835. https://doi.org/10.3390/ijms19030835.
53. Schmidt, M.; Schüler, S.C.; Hüttner, S.S.; von Eyss, B.; von Maltzahn, J. Adult Stem Cells at Work: Regenerating Skeletal Muscle. *Cell. Mol. Life Sci. CMLS* **2019**, *76*, 2559–2570. https://doi.org/10.1007/s00018-019-03093-6.
54. Yin, H.; Price, F.; Rudnicki, M.A. Satellite Cells and the Muscle Stem Cell Niche. *Physiol. Rev.* **2013**, *93*, 23–67. https://doi.org/10.1152/physrev.00043.2011.
55. Dumont, N.A.; Wang, Y.X.; Rudnicki, M.A. Intrinsic and Extrinsic Mechanisms Regulating Satellite Cell Function. *Dev. Camb. Engl.* **2015**, *142*, 1572–1581. https://doi.org/10.1242/dev.114223.
56. Chellini, F.; Tani, A.; Zecchi-Orlandini, S.; Sassoli, C. Influence of Platelet-Rich and Platelet-Poor Plasma on Endogenous Mechanisms of Skeletal Muscle Repair/Regeneration. *Int. J. Mol. Sci.* **2019**, *20*. https://doi.org/10.3390/ijms20030683.
57. Chatterjee, M.; Huang, Z.; Zhang, W.; Jiang, L.; Hultenby, K.; Zhu, L.; Hu, H.; Nilsson, G.P.; Li, N. Distinct Platelet Packaging, Release, and Surface Expression of Proangiogenic and Antiangiogenic Factors on Different Platelet Stimuli. *Blood* **2011**, *117*, 3907–3911. https://doi.org/10.1182/blood-2010-12-327007.
58. Brzoska, E.; Kowalewska, M.; Markowska-Zagrajek, A.; Kowalski, K.; Archacka, K.; Zimowska, M.; Grabowska, I.; Czerwińska, A.M.; Czarnecka-Góra, M.; Stremińska, W.; et al. Sdf-1 (CXCL12) Improves Skeletal Muscle Regeneration via the Mobilisation of Cxcr4 and CD34 Expressing Cells. *Biol. Cell* **2012**, *104*, 722–737. https://doi.org/10.1111/boc.201200022.
59. Salcedo, L.; Sopko, N.; Jiang, H.-H.; Damaser, M.; Penn, M.; Zutshi, M. Chemokine Upregulation in Response to Anal Sphincter and Pudendal Nerve Injury: Potential Signals for Stem Cell Homing. *Int. J. Colorectal Dis.* **2011**, *26*, 1577–1581. https://doi.org/10.1007/s00384-011-1269-6.
60. Andrews, V.; Sultan, A.H.; Thakar, R.; Jones, P.W. Occult Anal Sphincter Injuries--Myth or Reality? *BJOG Int. J. Obstet. Gynaecol.* **2006**, *113*, 195–200. https://doi.org/10.1111/j.1471-0528.2006.00799.x.
61. Rajasekaran, M.R.; Sinha, S.; Seo, Y.; Salehi, M.; Bhargava, V.; Mittal, R.K. Myoarchitectural and Functional Alterations in Rabbit External Anal Sphincter Muscle Following Experimental Surgical Trauma. *Am. J. Physiol. Gastrointest. Liver Physiol.* **2014**, *307*, G445–G451. https://doi.org/10.1152/ajpgi.00450.2013.
62. Cherry, D.A.; Rothenberger, D.A. Pelvic Floor Physiology. *Surg. Clin. North Am.* **1988**, *68*, 1217–1230. https://doi.org/10.1016/s0039-6109(16)44682-7.
63. Browning, G.G.; Motson, R.W. Anal Sphincter Injury. Management and Results of Parks Sphincter Repair. *Ann. Surg.* **1984**, *199*, 351–357. https://doi.org/10.1097/00000658-198403000-00017.
64. Walraven, M.; Hinz, B. Therapeutic Approaches to Control Tissue Repair and Fibrosis: Extracellular Matrix as a Game Changer. *Matrix Biol. J. Int. Soc. Matrix Biol.* **2018**, *71–72*, 205–224. https://doi.org/10.1016/j.matbio.2018.02.020.
65. Saldana Ruiz, N.; Kaiser, A.M. Fecal Incontinence—Challenges and Solutions. *World J. Gastroenterol.* **2017**, *23*, 11–24. https://doi.org/10.3748/wjg.v23.i1.11.
66. Frudinger, A.; Pfeifer, J.; Paede, J.; Kolovetsiou-Kreiner, V.; Marksteiner, R.; Halligan, S. Autologous Skeletal Muscle-Derived Cell Injection for Anal Incontinence Due to Obstetric Trauma: A Five-Year Follow-up of an Initial Study of Ten Patients. *Colorectal Dis. Off. J. Assoc. Coloproctology G. B. Irel.* **2015**. https://doi.org/10.1111/codi.12947.
67. Romaniszyn, M.; Rozwadowska, N.; Malcher, A.; Kolanowski, T.; Walega, P.; Kurpisz, M. Implantation of Autologous Muscle-Derived Stem Cells in Treatment of Fecal Incontinence: Results of an Experimental Pilot Study. *Tech. Coloproctology* **2015**, *19*, 685–696. https://doi.org/10.1007/s10151-015-1351-0.
68. Frudinger, A.; Marksteiner, R.; Pfeifer, J.; Margreiter, E.; Paede, J.; Thurner, M. Skeletal Muscle-Derived Cell Implantation for the Treatment of Sphincter-Related Faecal Incontinence. *Stem Cell Res. Ther.* **2018**, *9*, 233. https://doi.org/10.1186/s13287-018-0978-y.
69. Boyer, O.; Bridoux, V.; Giverne, C.; Bisson, A.; Koning, E.; Leroi, A.-M.; Chambon, P.; Déhayes, J.; Le Corre, S.; Jacquot, S.; et al. Autologous Myoblasts for the Treatment of Fecal Incontinence: Results of a Phase 2 Randomized Placebo-Controlled Study (MIAS). *Ann. Surg.* **2018**, *267*, 443–450. https://doi.org/10.1097/SLA.0000000000002268.
70. Araki, J.; Nishizawa, Y.; Fujita, N.; Sato, T.; Iizuka, T.; Kamata, M.; Hatayama, N.; Yakura, T.; Hirai, S.; Tashiro, K.; et al. Anorectal Transplantation: The First Long-Term Success in a Canine Model. *Ann. Surg.* **2021**. https://doi.org/10.1097/SLA.0000000000004141.
71. Sarveazad, A.; Newstead, G.L.; Mirzaei, R.; Joghataei, M.T.; Bakhtiari, M.; Babahajian, A.; Mahjoubi, B. A New Method for Treating Fecal Incontinence by Implanting Stem Cells Derived from Human Adipose Tissue: Preliminary Findings of a Randomized Double-Blind Clinical Trial. *Stem Cell Res. Ther.* **2017**, *8*, 40. https://doi.org/10.1186/s13287-017-0489-2.
72. De la Portilla, F.; Guerrero, J.L.; Maestre, M.V.; Leyva, L.; Mera, S.; García-Olmo, D.; Rodríguez, A.; Mata, R.; Lora, F. Treatment of Fecal Incontinence with Autologous Expanded Mesenchymal Stem Cells: Results of a Pilot Study. *Colorectal Dis. Off. J. Assoc. Coloproctology G. B. Irel.* **2020**. https://doi.org/10.1111/codi.15382.
73. Frudinger, A.; Kölle, D.; Schwaiger, W.; Pfeifer, J.; Paede, J.; Halligan, S. Muscle-Derived Cell Injection to Treat Anal Incontinence Due to Obstetric Trauma: Pilot Study with 1 Year Follow-Up. *Gut* **2010**, *59*, 55–61. https://doi.org/10.1136/gut.2009.181347.
74. Romaniszyn, M.; Michal, R.; Rozwadowska, N.; Natalia, R.; Nowak, M.; Marcin, N.; Malcher, A.; Agnieszka, M.; Kolanowski, T.; Tomasz, K.; et al. Successful Implantation of Autologous Muscle-Derived Stem Cells in Treatment of Faecal Incontinence Due to External Sphincter Rupture. *Int. J. Colorectal Dis.* **2013**, *28*, 1035–1036. https://doi.org/10.1007/s00384-013-1692-y.
75. Frenckner, B.; Euler, C.V. Influence of Pudendal Block on the Function of the Anal Sphincters. *Gut* **1975**, *16*, 482–489. https://doi.org/10.1136/gut.16.6.482.
76. Burleigh, D.E.; D’Mello, A. Neural and Pharmacologic Factors Affecting Motility of the Internal Anal Sphincter. *Gastroenterology* **1983**, *84*, 409–417.
77. Bitar, K.N.; Raghavan, S. Intestinal Tissue Engineering: Current Concepts and Future Vision of Regenerative Medicine in the Gut. *Neurogastroenterol. Motil. Off. J. Eur. Gastrointest. Motil. Soc.* **2012**, *24*, 7–19. https://doi.org/10.1111/j.1365-2982.2011.01843.x.
78. Szymanski, P.T.; Chacko, T.K.; Rovner, A.S.; Goyal, R.K. Differences in Contractile Protein Content and Isoforms in Phasic and Tonic Smooth Muscles. *Am. J. Physiol.* **1998**, *275*, C684-692. https://doi.org/10.1152/ajpcell.1998.275.3.C684.
79. Somara, S.; Gilmont, R.R.; Dennis, R.G.; Bitar, K.N. Bioengineered Internal Anal Sphincter Derived from Isolated Human Internal Anal Sphincter Smooth Muscle Cells. *Gastroenterology* **2009**, *137*, 53–61. https://doi.org/10.1053/j.gastro.2009.03.036.
80. Rattan, S.; Singh, J.; Kumar, S.; Phillips, B. Nature of Extracellular Signal That Triggers RhoA/ROCK Activation for the Basal Internal Anal Sphincter Tone in Humans. *Am. J. Physiol.-Gastrointest. Liver Physiol.* **2015**, *308*, G924–G933. https://doi.org/10.1152/ajpgi.00017.2015.
81. Rattan, S. The Internal Anal Sphincter: Regulation of Smooth Muscle Tone and Relaxation. *Neurogastroenterol. Motil. Off. J. Eur. Gastrointest. Motil. Soc.* **2005**, *17 Suppl 1*, 50–59. https://doi.org/10.1111/j.1365-2982.2005.00659.x.
82. Singh, J.; Boopathi, E.; Addya, S.; Phillips, B.; Rigoutsos, I.; Penn, R.B.; Rattan, S. Aging-Associated Changes in MicroRNA Expression Profile of Internal Anal Sphincter Smooth Muscle: Role of MicroRNA-133a. *Am. J. Physiol.-Gastrointest. Liver Physiol.* **2016**, *311*, G964–G973. https://doi.org/10.1152/ajpgi.00290.2016.
83. Singh, J.; Mohanty, I.; Addya, S.; Phillips, B.; Mee Yong, H.; An, S.S.; Penn, R.B.; Rattan, S. Role of Differentially Expressed MicroRNA-139-5p in the Regulation of Phenotypic Internal Anal Sphincter Smooth Muscle Tone. *Sci. Rep.* **2017**, *7*, 1477. https://doi.org/10.1038/s41598-017-01550-5.
84. Bitar, K.N. Aging and Gi Smooth Muscle Fecal Incontinence: Is Bioengineering an Option. *Exp. Gerontol.* **2005**, *40*, 643–649. https://doi.org/10.1016/j.exger.2005.04.008.
85. Townsend, M.K.; Matthews, C.A.; Whitehead, W.E.; Grodstein, F. Risk Factors for Fecal Incontinence in Older Women. *Am. J. Gastroenterol.* **2013**, *108*, 113–119. https://doi.org/10.1038/ajg.2012.364.
86. Yarandi, S.S.; Srinivasan, S. Diabetic Gastrointestinal Motility Disorders and the Role of Enteric Nervous System: Current Status and Future Directions. *Neurogastroenterol. Motil.* **2014**, *26*, 611–624. https://doi.org/https://doi.org/10.1111/nmo.12330.
87. Reszczyńska, M.; Kempiński, R. The Prevalence of Enteropathy Symptoms from the Lower Gastrointestinal Tract and the Evaluation of Anorectal Function in Diabetes Mellitus Patients. *J. Clin. Med.* **2021**, *10*, 415. https://doi.org/10.3390/jcm10030415.
88. Papathanasopoulos, A.; Van Oudenhove, L.; Katsanos, K.; Christodoulou, D.; Tack, J.; Tsianos, E.V. Severity of Fecal Urgency and Incontinence in Inflammatory Bowel Disease: Clinical, Manometric and Sonographic Predictors. *Inflamm. Bowel Dis.* **2013**, *19*, 2450–2456. https://doi.org/10.1097/MIB.0b013e3182a2952b.
89. Bassotti, G.; Antonelli, E.; Villanacci, V.; Nascimbeni, R.; Dore, M.P.; Pes, G.M.; Maconi, G. Abnormal Gut Motility in Inflammatory Bowel Disease: An Update. *Tech. Coloproctology* **2020**, *24*, 275–282. https://doi.org/10.1007/s10151-020-02168-y.
90. Hosokawa, T.; Konuma, N.; Ikeda, T.; Hashimoto, M.; Kaneda, H.; Ohashi, K.; Matsumoto, T.; Koshinaga, T. Establishment of a New Anal Sphincter Injury Model in Rats Based on Cardiotoxin. *J. Pediatr. Surg.* **2015**, *50*, 1352–1358. https://doi.org/10.1016/j.jpedsurg.2014.12.028.
91. Saihara, R.; Komuro, H.; Urita, Y.; Hagiwara, K.; Kaneko, M. Myoblast Transplantation to Defecation Muscles in a Rat Model: A Possible Treatment Strategy for Fecal Incontinence after the Repair of Imperforate Anus. *Pediatr. Surg. Int.* **2009**, *25*, 981–986. https://doi.org/10.1007/s00383-009-2454-3.
92. Craig, J.B.; Lane, F.L.; Nistor, G.; Motakef, S.; Pham, Q.-A.; Keirstead, H. Allogenic Myoblast Transplantation in the Rat Anal Sphincter. *Female Pelvic Med. Reconstr. Surg.* **2010**, *16*, 205–208. https://doi.org/10.1097/SPV.0b013e3181ec1edd.
93. Son, I.T.; Lee, H.S.; Ihn, M.H.; Lee, K.H.; Kim, D.-W.; Lee, K.-W.; Kim, J.-S.; Kang, S.-B. Isolation of Internal and External Sphincter Progenitor Cells from the Human Anal Sphincter with or without Radiotherapy. *Colorectal Dis. Off. J. Assoc. Coloproctology G. B. Irel.* **2019**, *21*, 38–47. https://doi.org/10.1111/codi.14351.
94. Singh, J.; Rattan, S. Bioengineered Human IAS Reconstructs with Functional and Molecular Properties Similar to Intact IAS. *Am. J. Physiol. Gastrointest. Liver Physiol.* **2012**, *303*, G713-722. https://doi.org/10.1152/ajpgi.00112.2012.
95. Gilmont, R.R.; Raghavan, S.; Somara, S.; Bitar, K.N. Bioengineering of Physiologically Functional Intrinsically Innervated Human Internal Anal Sphincter Constructs. *Tissue Eng. Part A* **2014**, *20*, 1603–1611. https://doi.org/10.1089/ten.TEA.2013.0422.
96. Raghavan, S.; Miyasaka, E.A.; Gilmont, R.R.; Somara, S.; Teitelbaum, D.H.; Bitar, K.N. Perianal Implantation of Bioengineered Human Internal Anal Sphincter Constructs Intrinsically Innervated with Human Neural Progenitor Cells. *Surgery* **2014**, *155*, 668–674. https://doi.org/10.1016/j.surg.2013.12.023.
97. Zakhem, E.; Rego, S.L.; Raghavan, S.; Bitar, K.N. The Appendix as a Viable Source of Neural Progenitor Cells to Functionally Innervate Bioengineered Gastrointestinal Smooth Muscle Tissues. *Stem Cells Transl. Med.* **2015**, *4*, 548–554. https://doi.org/10.5966/sctm.2014-0238.
98. Rego, S.L.; Raghavan, S.; Zakhem, E.; Bitar, K.N. Enteric Neural Differentiation in Innervated, Physiologically Functional, Smooth Muscle Constructs Is Modulated by Bone Morphogenic Protein 2 Secreted by Sphincteric Smooth Muscle Cells. *J. Tissue Eng. Regen. Med.* **2017**, *11*, 1251–1261. https://doi.org/10.1002/term.2027.
99. Raghavan, S.; Miyasaka, E.A.; Hashish, M.; Somara, S.; Gilmont, R.R.; Teitelbaum, D.H.; Bitar, K.N. Successful Implantation of Physiologically Functional Bioengineered Mouse Internal Anal Sphincter. *Am. J. Physiol.—Gastrointest. Liver Physiol.* **2010**, *299*, G430–G439. https://doi.org/10.1152/ajpgi.00269.2009.
100. Hashish, M.; Raghavan, S.; Somara, S.; Gilmont, R.R.; Miyasaka, E.; Bitar, K.N.; Teitelbaum, D.H. Surgical Implantation of a Bioengineered Internal Anal Sphincter. *J. Pediatr. Surg.* **2010**, *45*, 52–58. https://doi.org/10.1016/j.jpedsurg.2009.10.010.
101. Miyasaka, E.A.; Raghavan, S.; Gilmont, R.R.; Mittal, K.; Somara, S.; Bitar, K.N.; Teitelbaum, D.H. In Vivo Growth of a Bioengineered Internal Anal Sphincter: Comparison of Growth Factors for Optimization of Growth and Survival. *Pediatr. Surg. Int.* **2011**, *27*, 137–143. https://doi.org/10.1007/s00383-010-2786-z.
102. Bohl, J.L.; Zakhem, E.; Bitar, K.N. Successful Treatment of Passive Fecal Incontinence in an Animal Model Using Engineered Biosphincters: A 3-Month Follow-Up Study. *Stem Cells Transl. Med.* **2017**, *6*, 1795–1802. https://doi.org/10.1002/sctm.16-0458.
103. Raghavan, S.; Gilmont, R.R.; Miyasaka, E.A.; Somara, S.; Srinivasan, S.; Teitelbaum, D.H.; Bitar, K.N. Successful Implantation of Bioengineered, Intrinsically Innervated, Human Internal Anal Sphincter. *Gastroenterology* **2011**, *141*, 310–319. https://doi.org/10.1053/j.gastro.2011.03.056.
104. Tierney, M.T.; Sacco, A. Satellite Cell Heterogeneity in Skeletal Muscle Homeostasis. *Trends Cell Biol.* **2016**, *26*, 434–444. https://doi.org/10.1016/j.tcb.2016.02.004.
105. Thurner, M.; Deutsch, M.; Janke, K.; Messner, F.; Kreutzer, C.; Beyl, S.; Couillard-Després, S.; Hering, S.; Troppmair, J.; Marksteiner, R. Generation of Myogenic Progenitor Cell-Derived Smooth Muscle Cells for Sphincter Regeneration. *Stem Cell Res. Ther.* **2020**, *11*, 233. https://doi.org/10.1186/s13287-020-01749-w.
106. Tedesco, F.S.; Moyle, L.A.; Perdiguero, E. Muscle Interstitial Cells: A Brief Field Guide to Non-Satellite Cell Populations in Skeletal Muscle. *Methods Mol. Biol. Clifton NJ* **2017**, *1556*, 129–147. https://doi.org/10.1007/978-1-4939-6771-1_7.
107. Torrente, Y.; Belicchi, M.; Marchesi, C.; D’Antona, G.; Cogiamanian, F.; Pisati, F.; Gavina, M.; Giordano, R.; Tonlorenzi, R.; Fagiolari, G.; et al. Autologous Transplantation of Muscle-Derived CD133+ Stem Cells in Duchenne Muscle Patients. *Cell Transplant.* **2007**, *16*, 563–577.
108. Zhang, D.; Wang, L.; Zhang, F.; Li, C.; Zhu, T.; Cao, K.; Ma, W.; Yang, Z. Nine-Year Follow-up of Local Implantation of Autologous Skeletal Myoblasts in a Patient with Coronary Heart Disease. *Am. J. Case Rep.* **2013**, *14*, 139–142. https://doi.org/10.12659/AJCR.883903.
109. Lecoeur, C.; Swieb, S.; Zini, L.; Rivière, C.; Combrisson, H.; Ghérardi, R.; Abbou, C.; Yiou, R. Intraurethral Transfer of Satellite Cells by Myofiber Implants Results in the Formation of Innervated Myotubes Exerting Tonic Contractions. *J. Urol.* **2007**, *178*, 332–337. https://doi.org/10.1016/j.juro.2007.02.044.
110. Abbasi-Malati, Z.; Roushandeh, A.M.; Kuwahara, Y.; Roudkenar, M.H. Mesenchymal Stem Cells on Horizon: A New Arsenal of Therapeutic Agents. *Stem Cell Rev.* **2018**, *14*, 484–499. https://doi.org/10.1007/s12015-018-9817-x.
111. Sanabria-de la Torre, R.; Quiñones-Vico, M.I.; Fernández-González, A.; Sánchez-Díaz, M.; Montero-Vílchez, T.; Sierra-Sánchez, Á.; Arias-Santiago, S. Alloreactive Immune Response Associated to Human Mesenchymal Stromal Cells Treatment: A Systematic Review. *J. Clin. Med.* **2021**, *10*, 2991. https://doi.org/10.3390/jcm10132991.
112. Meier, R.P.H.; Müller, Y.D.; Morel, P.; Gonelle-Gispert, C.; Bühler, L.H. Transplantation of Mesenchymal Stem Cells for the Treatment of Liver Diseases, Is There Enough Evidence? *Stem Cell Res.* **2013**, *11*, 1348–1364. https://doi.org/10.1016/j.scr.2013.08.011.
113. Balaphas, A.; Meyer, J.; Sadoul, R.; Morel, P.; Gonelle-Gispert, C.; Bühler, L.H. Extracellular Vesicles: Future Diagnostic and Therapeutic Tools for Liver Disease and Regeneration. *Liver Int. Off. J. Int. Assoc. Study Liver* **2019**. https://doi.org/10.1111/liv.14189.
114. Meier, R.P.H.; Mahou, R.; Morel, P.; Meyer, J.; Montanari, E.; Muller, Y.D.; Christofilopoulos, P.; Wandrey, C.; Gonelle-Gispert, C.; Bühler, L.H. Microencapsulated Human Mesenchymal Stem Cells Decrease Liver Fibrosis in Mice. *J. Hepatol.* **2015**, *62*, 634–641. https://doi.org/10.1016/j.jhep.2014.10.030.
115. Pokrywczynska, M.; Jundzill, A.; Warda, K.; Buchholz, L.; Rasmus, M.; Adamowicz, J.; Bodnar, M.; Marszalek, A.; Helmin-Basa, A.; Michalkiewicz, J.; et al. Does the Mesenchymal Stem Cell Source Influence Smooth Muscle Regeneration in Tissue-Engineered Urinary Bladders? *Cell Transplant.* **2017**, *26*, 1780–1791. https://doi.org/10.1177/0963689717722787.
116. Zhang, N.; Qin, X.; Zhang, J.; Zhang, Z.; Li, Y.; Xie, Y.; Kong, D.; Du, R.; Huang, X.; Xu, Y. Bone Marrow Mesenchymal Stem Cells Accelerate the Morphological and Functional Recovery of Neovaginas. *Artif. Organs* **2018**, *42*, 1206–1215. https://doi.org/10.1111/aor.13297.
117. Nakamura, Y.; Miyaki, S.; Ishitobi, H.; Matsuyama, S.; Nakasa, T.; Kamei, N.; Akimoto, T.; Higashi, Y.; Ochi, M. Mesenchymal-Stem-Cell-Derived Exosomes Accelerate Skeletal Muscle Regeneration. *FEBS Lett.* **2015**, *589*, 1257–1265. https://doi.org/10.1016/j.febslet.2015.03.031.
118. Linard, C.; Brachet, M.; L’homme, B.; Strup-Perrot, C.; Busson, E.; Bonneau, M.; Lataillade, J.-J.; Bey, E.; Benderitter, M. Long-Term Effectiveness of Local BM-MSCs for Skeletal Muscle Regeneration: A Proof of Concept Obtained on a Pig Model of Severe Radiation Burn. *Stem Cell Res. Ther.* **2018**, *9*, 299. https://doi.org/10.1186/s13287-018-1051-6.
119. Hecker, L.; Baar, K.; Dennis, R.G.; Bitar, K.N. Development of a Three-Dimensional Physiological Model of the Internal Anal Sphincter Bioengineered in Vitro from Isolated Smooth Muscle Cells. *Am. J. Physiol. Gastrointest. Liver Physiol.* **2005**, *289*, G188-196. https://doi.org/10.1152/ajpgi.00335.2004.
120. Dominici, M.; Le Blanc, K.; Mueller, I.; Slaper-Cortenbach, I.; Marini, F.; Krause, D.; Deans, R.; Keating, A.; Prockop, D.; Horwitz, E. Minimal Criteria for Defining Multipotent Mesenchymal Stromal Cells. The International Society for Cellular Therapy Position Statement. *Cytotherapy* **2006**, *8*, 315–317. https://doi.org/10.1080/14653240600855905.
121. Gräs, S.; Tolstrup, C.K.; Lose, G. Regenerative Medicine Provides Alternative Strategies for the Treatment of Anal Incontinence. *Int. Urogynecol. J.* **2017**, *28*, 341–350. https://doi.org/10.1007/s00192-016-3064-y.
122. Baldari, S.; Di Rocco, G.; Piccoli, M.; Pozzobon, M.; Muraca, M.; Toietta, G. Challenges and Strategies for Improving the Regenerative Effects of Mesenchymal Stromal Cell-Based Therapies. *Int. J. Mol. Sci.* **2017**, *18*. https://doi.org/10.3390/ijms18102087.
123. Balaphas, A.; Schiltz, B.; Liot, E.; Robert-Yap, J.; Ris, F. What Is the Role of Stem Cell Therapy in the Treatment of Anal Incontinence? *Colorectal Dis. Off. J. Assoc. Coloproctology G. B. Irel.* **2021**, *23*, 551–552. https://doi.org/10.1111/codi.15433.
124. Bols, E.M.J.; Hendriks, H.J.M.; Berghmans, L.C.M.; Baeten, C.G.M.I.; de Bie, R.A. Responsiveness and Interpretability of Incontinence Severity Scores and FIQL in Patients with Fecal Incontinence: A Secondary Analysis from a Randomized Controlled Trial. *Int. Urogynecol. J.* **2013**, *24*, 469–478. https://doi.org/10.1007/s00192-012-1886-9.
125. Voorham-van der Zalm, P.J.; Voorham, J.C.; van den Bos, T.W.L.; Ouwerkerk, T.J.; Putter, H.; Wasser, M.N.J.M.; Webb, A.; DeRuiter, M.C.; Pelger, R.C.M. Reliability and Differentiation of Pelvic Floor Muscle Electromyography Measurements in Healthy Volunteers Using a New Device: The Multiple Array Probe Leiden (MAPLe). *Neurourol. Urodyn.* **2013**, *32*, 341–348. https://doi.org/10.1002/nau.22311.
126. Johnson, E.; Carlsen, E.; Steen, T.B.; Backer Hjorthaug, J.O.; Eriksen, M.T.; Johannessen, H.-O. Short- and Long-Term Results of Secondary Anterior Sphincteroplasty in 33 Patients with Obstetric Injury. *Acta Obstet. Gynecol. Scand.* **2010**, *89*, 1466–1472. https://doi.org/10.3109/00016349.2010.519019.
127. Lamblin, G.; Bouvier, P.; Damon, H.; Chabert, P.; Moret, S.; Chene, G.; Mellier, G. Long-Term Outcome after Overlapping Anterior Anal Sphincter Repair for Fecal Incontinence. *Int. J. Colorectal Dis.* **2014**, *29*, 1377–1383. https://doi.org/10.1007/s00384-014-2005-9.
128. Garcia, S.; Bernad, A.; Martín, M.C.; Cigudosa, J.C.; Garcia-Castro, J.; de la Fuente, R. Pitfalls in Spontaneous in Vitro Transformation of Human Mesenchymal Stem Cells. *Exp. Cell Res.* **2010**, *316*, 1648–1650. https://doi.org/10.1016/j.yexcr.2010.02.016.
129. Torsvik, A.; Røsland, G.V.; Svendsen, A.; Molven, A.; Immervoll, H.; McCormack, E.; Lønning, P.E.; Primon, M.; Sobala, E.; Tonn, J.-C.; et al. Spontaneous Malignant Transformation of Human Mesenchymal Stem Cells Reflects Cross-Contamination: Putting the Research Field on Track - Letter. *Cancer Res.* **2010**, *70*, 6393–6396. https://doi.org/10.1158/0008-5472.CAN-10-1305.
130. Trébol, J.; Carabias-Orgaz, A.; García-Arranz, M.; García-Olmo, D. Stem Cell Therapy for Faecal Incontinence: Current State and Future Perspectives. *World J. Stem Cells* **2018**, *10*, 82–105. https://doi.org/10.4252/wjsc.v10.i7.82.
131. Lipsitz, Y.Y.; Milligan, W.D.; Fitzpatrick, I.; Stalmeijer, E.; Farid, S.S.; Tan, K.Y.; Smith, D.; Perry, R.; Carmen, J.; Chen, A.; et al. A Roadmap for Cost-of-Goods Planning to Guide Economic Production of Cell Therapy Products. *Cytotherapy* **2017**, *19*, 1383–1391. https://doi.org/10.1016/j.jcyt.2017.06.009.
132. De la Portilla, F. Reply to Balaphas et Al. *Colorectal Dis. Off. J. Assoc. Coloproctology G. B. Irel.* **2021**, *23*, 1002–1003. https://doi.org/10.1111/codi.15527.

The authors apologize for any inconvenience caused and state that the scientific conclusions are unaffected. This correction was approved by the academic editor. The original publication has also been updated.

## References

[1] Balaphas A., Meyer J., Meier R.P.H., Liot E., Buchs N.C., Roche B., Toso C., Bühler L.H., Gonelle-Gispert C., Ris F. (2021). Cell Therapy for Anal Sphincter Incontinence: Where Do We Stand?. Cells.

