# Supplementary material for: Cell Therapy for Anal Sphincter Incontinence: Where Do We Stand?
Source: Cells. 2021 Aug 13;10(8):2086. doi: 10.3390/cells10082086 (PMC8394955; doi:10.3390/cells10082086)
Supplement: Supplementary file 1 [file cells-10-02086-s001.zip › cells-1300408-supplementary.pdf]

Table S1. List and description of identified *in vivo* publications investigating cell therapy for the treatment of anal sphincter incontinence.

Publication	Short description	*n*	Species	Concerned sphincter	Cellular therapy	Number of injected cell	Main outcomes	Follow-up from cell therapy to outcome	Suggesting positive effect on ASI
Li et al. 2020	Compare im versus iv injection of bone-marrow PC after sphincter excision	96	Rat	IAS/EAS	Allogeneic transplantation of bone-marrow derived progenitor cells	10 mio	Histology, anorectal manometry, electromyography, IF	28 days	Yes
Dadhich et al. 2019	Development of a clinically relevant model of ASI; harvesting of IAS SMC PC and NPC; generation and implantation of biosphincters	10	NHP	IAS	Biosphincters made from IAS SMC PC and enteric NPC	NA	Histology, anorectal manometry, IHC and IF	12 months	Yes
Sarveazad et al. 2019	Assessment of adipose tissue PC and laser treatment directly after sphincterotomy	35	Rabbit	IAS/EAS	Xenotransplantation of human adipose tissue PC	2 mio	Histology, anorectal manometry, electromyography, IF	12 weeks	Yes
Inoue et al. 2018	Assessment of sheets of adipose tissue PC transplanted directly after sphincterotomy	18	Rat	IAS/EAS	Allogeneic transplantation of adipose tissue PC sheets	NA	Histology, anal manometry, IHC and IF	28 days	Yes
Kuismanen et al. 2018	Assessment of human adipose tissue PC in saline solution or in polyacrylamide hydrogel injected immediately after sphincter injury compared to saline/hydrogel injection.	60	Rat	IAS/EAS	Human adipose tissue PC injected into both ends of EAS	0.3 mio	Histology, anal manometry, µCT imaging	2, 4 weeks	Yes
Li et al. 2018	Assessment of electroacupuncture on the homing of bone-marrow PC injected intravenously or into anal sphincters lesion compared to controls without injury, without electroacupuncture or without cell injection	60	Rat	IAS/EAS	Syngeneic bone-marrow PC injected into the tail vein	9.6 mio	Histology, qPCR	1, 3, 7, 14 days	Yes
Trébol et al. 2018	Comparison of labelled adipose tissue PC injection, adipose tissue PC seeded suture with sphincteroplasty or sphincteroplasty with normal	36	Rat	IAS/EAS	Injection of cells into muscle edges or sphincter repair with MSC seeded suture	1-3 mio	Histology	1, 4, 7days	No

	suture followed by labelled adipose tissue PC injection								
Bohl et al. 2017	Randomized controlled study to compare the effect of bioengineered autologous IAS sphincter implantation to sham implantation or no surgery at all.	20	Rabbit	IAS	Autologous biosphincters from IAS SMC PC and enteric NPC	2 mio SMC PC and 0.8mio NPC	Anal manometry	12 weeks	Yes
Sun et al. 2017	Randomized controlled, comparison of MSC with scaffold to MSC with SDF-1 plasmid or SDF-1 plasmid alone	32	Rat	IAS and EAS	Injection of syngeneic bone marrow MSC at the end of the defect	0.8 mio	Anal manometry (basal tone)	4 weeks	Yes
Sun et al. 2017	Randomized controlled study comparing bone-marrow PC with scaffold to bone-marrow PC with SDF-1 plasmid or SDF-1 plasmid alone	32	Rat	IAS/EAS	Injection of syngeneic bone marrow PC at the end of the defect	0.8 mio	Anal manometry	8 weeks	Yes
Sun et al. 2016	Evaluate the effect of electrical stimulation on bone marrow PC implantation and bone marrow PC homing.	58	Rat	IAS/EAS	Injection of syngeneic bone marrow PC at the end of the defect	1-2 mio	Anal manometry histology	4 weeks	Yes
Ding et al. 2016	Randomized controlled comparing end-to-end sphincteric repair versus end-to-end sphincteric repair with acellular dermal matrix versus end-to-end sphincteric repair with acellular dermal matrix seeded with bone marrow PC versus end-to-end sphincteric repair with acellular dermal matrix seeded with bone marrow PC C overexpressing galectin-1	-	Rat	IAS/EAS	Acellular dermal matrix with syngeneic bone marrow PC transfected with galectin-1 gene and sphincteroplasty	-	Histology	6 weeks	Yes
Mazzanti et al. 2016	Comparison between minimally manipulated bone marrow-derived mononuclear cell and bone marrow PC	32	Rat	IAS/EAS	Injection of syngeneic bone marrow PC or bone marrow-derived mononuclear cells at the ends of the defect	3 mio	Histology, physiological functional evaluation, morphometric	4 weeks	Yes
Kajbafzadeh et al. 2016	Comparing techniques of transplantation of decellularized external sphincter matrix with	16	Rabbit	EAS	Injection of autologous myogenic satellite cells from quadriceps into	70 mio	Histology	104 weeks	Yes

	and without injection of myogenic satellite cells.				transplanted allogeneic decellularized EAS				
Oh et al. 2015	Randomized controlled study comparing of muscle tissue PC injection with polycaprolactone beads versus no treatment	15	Dog	IAS/EAS	Injection into injury of autologous muscle tissue PC from hindlimb seeded in polycaprolactone beads	10 mio	Anal manometry (basal tone and contractile tone)	12 weeks	Yes
Oh et al. 2015	Randomized controleld study comparing muscle tissue PC injection to fibroblast growth factor-loaded polycaprolactone beads versus no treatment	10	Dog	IAS/EAS	Injection into injury of autologous muscle tissue PC from hindlimb seeded in polycaprolactone beads	60 mio	Histology, anal manometry, electrophysiology	4 or 12 weeks	Yes
Montoya et al. 2015	Assessment of PEG-based hydrogel seeded myoblasts compared to a bulking agent	80	Rat	IAS/EAS	Re-exposition of sphincter edges and injection of commercial H9c2 rat heart myoblasts seeded in a PEG-based hydrogel	3.2 mio	Histology, electrophysiology, muscle volume	4 or 12 weeks	Yes
Fitzwater et al. 2015	Assessment of myoblast injection before sphincteroplasty versus sphincteroplasty alone	40	Rat	IAS/EAS	Injection of commercial H9c2 rat heart myoblasts before sphincteroplasty	3.2 mio	Histology, muscle volume	1 or 12 weeks	No
Salcedo et al. 2014	Assessment of bone-marrow PC local injection 24hours or 3 weeks after injury and bone-marrow PC homing 3 weeks after intravenous injection compared to control without injury but bone-marrow PC injection	50	Rat	IAS/EAS	Injection of syngeneic bone-marrow PC into injury	-	Histology, anal manometry	10 days or 5 weeks	Yes
Raghavan et al. 2014	Preliminary report on heterotopic transplantation of human bioengineered internal sphincter in healthy athymic rats	-	Rat	IAS	Non-orthotopic transplantation of bioengineered internal sphincter with human SMC PC from IAS and human	0.5 mio SMC PC along with 0.2 enteric NPC	Histology, physiological functional evaluation	4 weeks	NA

					enteric NPCs from human rectum/colon				
Elmi et al. 2014	Assessment of the fate of sphincter transplanted labelled muscle tissue PC with MRI tracking	12	Rabbit	IAS/EAS	Autologous muscle tissue PC from quadriceps injected at the site of damage	90 mio	Anal manometry, EMG, MRI	1 hour, 1 week, 2 weeks, 4 weeks	Yes
Bisson et al. 2013	Preliminary experiment on muscle tissue PC injection compared to saline injection	-	Rat	IAS/EAS	Syngeneic injection of muscle tissue PC at the site of injury and into its borders	0.3-30 millions	Histology, Anal manometry	4 or 6 weeks	Yes
Jacobs et al. 2013	Assessment of the safety of muscle tissue PC transplantation into anal sphincters	33	Rat	IAS/EAS	Syngeneic injection of muscle tissue PC from young rat hindlimbs into the damaged EAS only, followed by sphincteroplasty	5 mio	Histology	4 weeks	NA
Kang et al. 2013	Preliminary experiment on injection of polycaprolactone beads containing muscle tissue PC s after sphincter injury compared to beads alone	10	Dog	IAS/EAS	Injection into injury of autologous myoblasts from gastrocnemius muscle seeded in polycaprolactone beads	-	Histology, Anal manometry, electrophysiology	12 weeks	No
Lane t al. 2013	Assessment of myoblasts injection during sphincteroplasty for a grade four episiotomy	33	Rat	IAS/EAS	Syngeneic injection of muscle tissue PC from young rat hindlimbs into the damaged EAS only, followed by sphincteroplasty	5 mio	Anal manometry, EMG	1 to 4 weeks	Yes
Salcedo et al. 2013	Assessment of local bone-marrow PC injection 24hours after sphincter transection or puddendal nerve crushing compared to sham lesion and/or saline injection	70	Rat	IAS/EAS	Injection of syngeneic bone-marrow PC into injury	2 mio	Histology, anal manometry, EMG	10 days	Yes
Pathi et al. 2012	Assessment of local versus iv injection of bone-marrow PC controlled with PBS injection or no lesion	224	Rat	IAS/EAS	Injection of syngeneic bone-marrow PC into injury or in the tail vein immediately after injury	4 mio	Histology, physiological functional evaluation, RT-PCR	1day, 2 days 7days, 21days	Yes
Raghavan et al. 2011	Assessment of the functionality of human bioengineered circular	-	Mouse	IAS	Transplantation of construct under the	0.1mio mouse fetal	Histology, IHC,	4 weeks	NA

	sphincteric smooth muscle cell constructs seeded with mouse fetal enteric neurons (commercial) implantated heterotopically in healthy mice				skin of *RAG1* ko mice with FGF pump	enteric neurons, 0.4 mio SMC PC	physiological functionality evaluation		
Craig et al 2010	Assessment of the fate of muscle tissue PC transplanted into EAS	4	Rat	EAS	Injection of cells into the EAS under electromyographic guidance	1.5-4.5 mio	Histology	10days	NA
Hashish et al. 2010	Feasibility of bioengineered mouse circular sphincteric smooth muscle cell constructs heterotopically implanted in healthy mice	10	Mouse	IAS	Transplantation of construct under the skin of mice with FGF pump	-	Histology	4 weeks	NA
Raghavan et al. 2010	Assessment of the functionality of mouse bioengineered circular sphincteric smooth muscle cell constructs with heterotopic implantation in healthy mice	1	Mouse	IAS	Transplantation of construct under the skin of mice with FGF pump	0.1 mio	Histology, IF, physiological functionality evaluation, western blot	4 weeks	NA
Kajbafzadeh et al. 2010	Feasibility study of local muscle tissue PC injection 3 weeks after sphincter injury compared to saline solution injection	21	Rabbit	EAS	Injection into the defect of quadriceps tissue autologous PC	70 mio	Histology, anal manometry, EMG	2, 4, 15 weeks	Yes
Miyasaka et al. 2011	Assessment of continuous infusion of growth factors on the development of a heterotopically transplanted bioengineered circular sphincteric smooth muscle cell compared to transplantation without growth factors pump	8	Mouse	IAS	Transplantation of construct under the skin of mice with growth factors pump	-	Histology, IF, physiological functionality evaluation	4 weeks	NA
White et al. 2010	Assessment of the effect of myoblast injection along with sphincteroplasty immediately after sphincterotomy controlled with sphincteroplasty alone or no reparation with or without cells injection	120	Rat	IAS/EAS	Injection of commercial H9c2 rat heart myoblasts before sphincteroplasty	3.2 mio	physiological functionality evaluation	1, 3 ,12weeks	Yes
Aghaee-Afshar et al. 2009	Assessment of rabbit bone-marrow PC or human umbilical cord matrix stem cells injections	31	Rabbit	EAS	Injection of rabbit bone-marrow PC or human umbilical cord	10’000	Histology, EMG	2 weeks	Yes

	after external sphincter section compared to saline/cell medium injection				matrix stem into injury with immunosuppression				
Saihara et al. 2009	Assessment of the fate of GFP labeled muscle tissue PC transplanted into the levator ani	-	Ra	Levator ani	Skin incision and exposition of levator ani for Injection of GFP labelled muscle tissue PC s into it	20 mio	Histology	1, 2, 4 weeks	NA
Kang et al. 2008	Assessment of rat muscle tissue PC injection directly after cryoinjury compared to control without injection or without injury nor injection	15	Rat	EAS	Injection of syngeneic gastrocnemius muscle muscle tissue PC into injury	3 mio	Histology, physiological functionality evaluation	1 week	No
Lorenzi et al. 2008	Preliminary study on injection of bone-marrow PC injection along with sphincteroplasty versus sham surgery or surgery with saline injection	24	Rat	IAS/EAS	Injection of syngeneic bone marrow PC at the end of the defect	1.5 mio	Histology, physiological functionality evaluation	4 weeks	Yes

ASI: anal sphincter incontinence, EAS: External anal sphincter, IAS: Internal anal sphincter, IF: Immunofluorescence, im: intramuscular, iv:intravenous, NA: Not applicable, NPC: non-progenitor cells, EMG: Electromyography, FGF-2: Fibroblast growth factor MSC: Mesenchymal Stem Cells, µCT: micro computed tomography, mio:million PC: progenitor cells, PEG: polyethylene glycol, SMC: smooth muscle cells

Table S2. List of *in vitro* publications. EAS: External anal sphincter, IAS: Internal anal sphincter.

Publication	Aim/design	Species source	Concerned sphinctered	Construct	Number of injected cell	Outcomes	Results
Son et al. 2019	Assess isolation of IAS and EAS after oncological surgery of the rectum and the effect of radiotherapy on the quality of isolation	Human	IAS/ EAS	NA	NA	-Immunofluorescence<br>-Western blot<br>-rtPCR<br>-Viability assay	Confirmation of expression profile. Radiotherapy affect only cell viability
Zakhem et al. 2015	Assess the potential of appendix-derived enteric neuronal progenitor cells on bioengineered sphincteric smooth muscle cell constructs	Rabbit	IAS	Sylgard, two separated layer in a gel of collagen/laminin	200'000 enteric neuronal progenitor cells with 500'000 sphincteric smooth muscle cell, initially	-Immunohistochemistry for neural and glial markers<br>-Western blot for neural and glial proteins expression and neurotransmitters<br>-Physiological functionality evaluation	Appendix-derived enteric neuronal progenitor cells are similar to small intestine-derived enteric neuronal progenitor cells
Rego et al. 2015	Assess the production of growth factors by bioengineered circular sphincteric smooth muscle cell constructs seeded with enteric neuronal progenitor cells and their contractility propriety compared to non-sphincteric constructs	Rabbit	IAS	Sylgard, two separated layer in a gel of collagen/laminin	200'000 enteric neuronal progenitor cells with 500'000 sphincteric smooth muscle cell, initially	-Immunohistochemistry for neural and glial markers<br>-Growth factors level assessment<br>-Physiological functionality evaluation	Increased neural progenitor cells differentiation, physiological response to stimulation (contraction) and levels of BMP2 protein in sphincteric constructs compared to non sphincteric constrcuts
Gilmont et al. 2014	Assess the feasibility of a bioengineered circular sphincteric smooth muscle cell constructs seeded with enteric neuronal progenitor cells, both of human origin	Human	IAS	Sylgard, two separated layer in a gel of collagen/laminin	200'000 enteric neuronal progenitor cells with 500'000 sphincteric smooth muscle cell, initially	-PCR<br>-Immunohistochemistry<br>-Physiological functionality evaluation	RNA for protein of contractile muscle, physiological response to stimulation (contraction)
Singh and Rattan 2012	Preliminary study with human bioengineered circular sphincteric smooth muscle cell constructs	Human	IAS	Sylgard, gel with collagen	10 millions, initially	-Physiological functionality evaluation<br>-Western blot<br>-Immunohistochemistry	Spontaneous basal tone, contraction following stimulation, Rho/ROCK dependence,

Somara et al. 2009	Preliminary study with human bioengineered circular sphincteric smooth muscle cell constructs	Human	IAS	Sylgard, loose fibrin gel	-	-Physiological functionality evaluation<br>-Western blot	Spontaneous basal tone, contraction following stimulation, increased expression of PKCα, RHoA, CPI-17 and HSP27 compared to other smooth muscle cells
Hecker et al. 2005	Preliminary study with rabbit bioengineered circular sphincteric smooth muscle cell constructs	Rabbit	IAS	Sylgard, loose fibrin gel	-	-Physiological functionality evaluation	Contraction following stimulation
